# The impact of mentoring relationships on professional identity formation in medical education: a systematic review

**DOI:** 10.1186/s12909-025-07158-y

**Published:** 2025-04-19

**Authors:** Lalit Kumar Radha Krishna, Nila Ravindran, Hannah Yi Fang Kwok, Xuan Yu Tan, Jasper Soh, Elizabeth Yong Mei Leong, Darius Wei Jun Wan, Tiat Yan Low, Aiden Wei-Jun Chan, Nicholas Chong Jin Lim, Yen Kit Ng, Arthena Anushka Thenpandiyan, Jun Rey Leong, Adele Yi Dawn Lim, Elaine Li Ying Quah, Leia Ning Tse, Sriram PL, Sri Priyanka Rajanala, Jun Kiat Lua, Varsha Rajalingam, Victoria Jia En Fam, Ranitha Govindasamy, Nur Amira Binte AbdulHamid, Crystal Lim, Eng Koon Ong, Shin Wei Sim, Stephen Mason, Simon Yew Kuang Ong

**Affiliations:** 1 Duke-NUS Medical School, National University of Singapore, 8 College Road, Singapore, 169857 Singapore; 2 Centre for Biomedical Ethics, National University of Singapore, Block MD11, 10 Medical Drive, #02- 03, Singapore, 117597 Singapore; 3 Division of Supportive and Palliative Care, National Cancer Centre Singapore, 30 Hospital Boulevard, Singapore, 168583 Singapore; 4 Division of Cancer Education, National Cancer Centre Singapore, 30 Hospital Boulevard, Singapore, 168583 Singapore; 5 Yong Loo Lin School of Medicine, National University of Singapore, NUHS Tower Block, Level 11, Block 1E, Kent Ridge Road, Singapore, 119228 Singapore; 6 Lee Kong Chian School of Medicine, Nanyang Technological University, 11 Mandalay Road, Singapore, 308207 Singapore; 7 Palliative Care Institute Liverpool, Academic Palliative & End of Life Care Centre, Cancer Research Centre, University of Liverpool, 200 London Road, Liverpool, L3 9TA UK; 8 Health Data Science, University of Liverpool, Whelan Building, The Quadrangle, Brownlow Hill, Liverpool, Liverpool, L69 3GB UK; 9 The Palliative Care Centre for Excellence in Research and Education, PalC, Dover Park Hospice, 10 Jalan Tan Tock Seng, Singapore, 308436 Singapore; 10 SingHealth Internal Medicine Residency, Singapore General Hospital, Outram Road, Singapore, 169608 Singapore; 11 Division of Psychosocial Oncology, National Cancer Centre Singapore, 30 Hospital Boulevard, Singapore, 168583 Singapore; 12 Medical Social Services, Singapore General Hospital, Block 3, Singapore, 169854 Singapore; 13 Division of Medical Oncology, National Cancer Centre Singapore, 30 Hospital Boulevard, Singapore, 168583 Singapore; 14 Geylang Polyclinic (National Healthcare Group Polyclinics), 21 Geylang East Central, Singapore, 389707 Singapore

**Keywords:** Mentoring relationships, Mentoring, Medical schools, Medicine, Professional identity formation, Personhood, Community of practice, Socialisation process

## Abstract

**Background:**

The promise that enduring and personalised mentoring relationships shape how mentees think, feel and act as professionals, or their professional identity formation (PIF), and thus how they interact, care and support patients and families has garnered significant interest. However, efforts to marshall these elements have been limited due to a lack of effective understanding. To address this lacunae, a systematic scoping review was carried out to map current knowledge on mentoring relationships and its impact on PIF.

**Methods:**

Guided by PRISMA guidelines and the Systematic Evidence-Based Approach (SEBA) to ensure a consistent and reproducible review, independent searches and appraisals of relevant articles published between 1st January 2000 and 4th December 2024 on PubMed, Embase, ERIC and Scopus databases were performed. Data from included articles were content and thematically analysed. Related themes and categories were combined using the SEBA methodology.

**Results:**

248 articles were identified across four databases and snowballing of key articles. A total of 27 articles were included. The domains identified were: (1) the mentoring ecosystem; (2) mentoring dynamics; (3) shifts in belief systems and professional identity; and (4) complex adaptive systems.

**Conclusions:**

The mentoring programme can be seen as a mentoring ecosystem, functioning as a community of practice and supporting the socialisation process within its boundaries and along the mentoring trajectory. The culture and structure of the mentoring ecosystem help inculcate the shared belief systems and programme identity. It also nurtures stakeholder investment and commitment, as well as their internal compass which is key to contending with the complex array of influences upon their development. Through the lens of a complex adaptive system, it is also possible to appreciate transitions between roles and responsibilities and the notion of being and becoming. These findings underline the evolving nature of practice and the need for personalised and longitudinal mentoring support.

**Supplementary Information:**

The online version contains supplementary material available at 10.1186/s12909-025-07158-y.

## Background

The success of mentoring in shaping how mentees “*think*,* act*,* and feel like a physician*” [1], or their professional identity formation (PIF) [2], has been attributed to its ability to nurture personalised and enduring mentoring relationships [3–6]. If mentoring and learning relationships are key to fostering effective PIF, understanding how such relationships are able to achieve this feat would be essential to the design of education initiatives beyond the mentoring sphere.

With new data highlighting the impact of maturing mentoring relationships influenced by the mentoring environment [3, 5, 7, 8], a better appreciation of the complex nature of mentoring relationships is required to enhance the design, knowledge, skill and assessment practices in mentoring and wider educational practices.

Here, the conceptualisation of mentorship and mentorship programmes within medical education is integral to this review. Mentorship is defined as *“a dynamic*,* context-sensitive relationship rooted in shared professional and personal interests*,* in which an experienced individual supports the growth of a less experienced mentee*,* fostering development and enrichment for both mentor and mentee”* [9]. Mentorship programmes, typically overseen by host organizations, are referred to as structured, goal-oriented initiatives designed to support the deliberate development of mentees through the guided transfer of knowledge, skills and values [10].

### Theoretical Lens

A constructivist ontological and relativist epistemological position was adopted in recognition of mentoring relationship existing as a social construct, influenced by both individual and contextual considerations [11–13]. These considerations are detailed in Table 1 below.

**Table 1:** Individual and contextual considerations

Individualised	Contextual
• Working styles, opportunities [14], attitudes, emotions, experience, skills, goals, demographic [15, 16], socio-cultural [17–19] and psycho-emotional features [15] • The physician’s meaning-making on the background of their belief systems, adaptation, and development; the importance placed on an interaction or specific incident; level of resilience and psycho-emotional status [11, 20]; and the available support that impact their responses [21–23]	• The mentoring programme’s setting in a formal or informal curriculum, working hours [24], rules [25], disciplinary consequences [26], programmes [27, 28], attention to PIF [29–31], administrative support [33], faculty training and evaluation [32, 33], access to personalised support and communication networks, hidden curriculum [30, 34–43], prevailing discourses [38, 44–47], daily activities [36, 48, 49], and rites of passage [1, 41, 43, 50–55] (*curricula determined by host organisations*). • The programme’s learning objectives [56], goals [57, 58], timelines and professional standards [59, 60], codes of conduct, expectations [61, 62], implicit norms [63], culture [64], sociocultural norms and legal requirements [65–68] • Accessible communication and flexible and personalised longitudinal support [3, 5–7, 12, 20, [69–77]

The influence of these cultural and contextual considerations reshapes individual belief systems within the four key domains of personhood, or ‘what makes you, you’. The Ring Theory of Personhood (RToP), a clinically evidenced framework [78, 79], and the Krishna-Pisupati Model of Professional Identity Formation (KPM) [3, 5, 11] were used to sketch changes in identity critical to efforts in supporting, guiding, assessing and overseeing progress and PIF.

### The ring theory of personhood

**Fig. 1:**
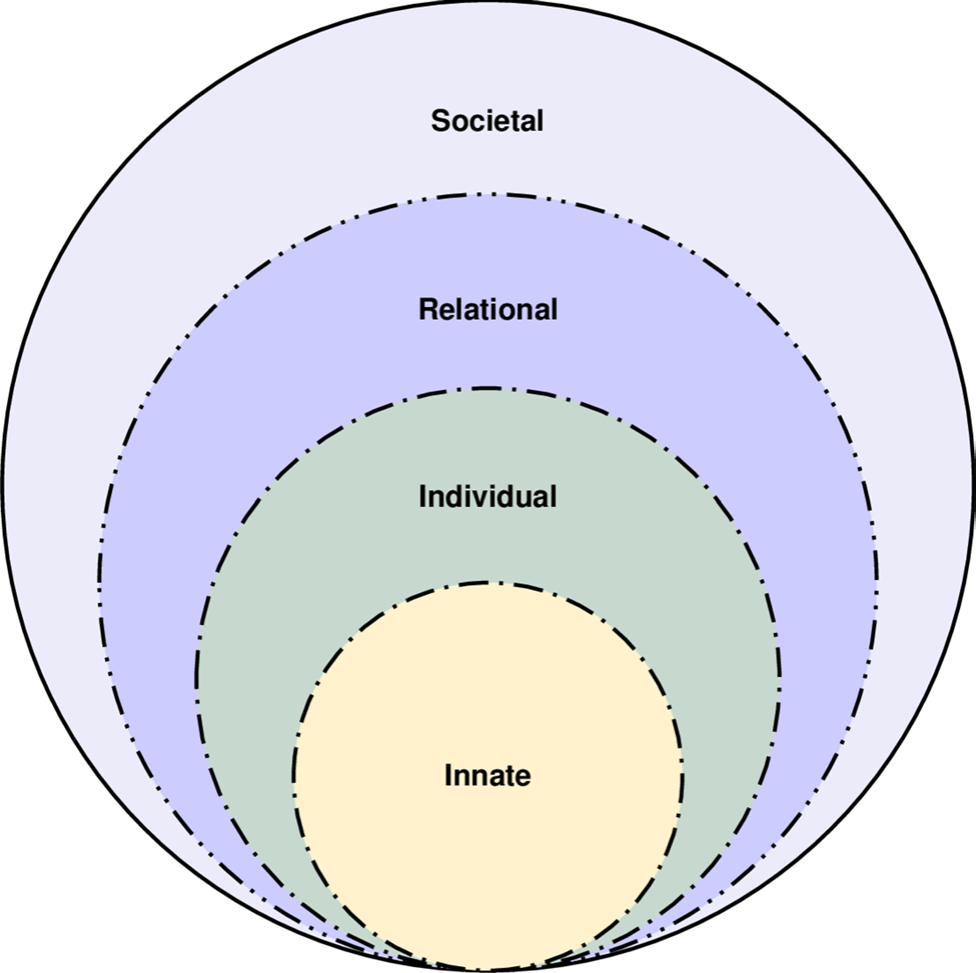
The Ring Theory of Personhood

The RToP considers the experiences of one stakeholder. Most often, this is the mentee’s perspective on changes in their Innate, Individual, Relational and Societal belief systems which reflect changes in their self-identity [3, 5] (Fig. 1).

The RToP suggests that a better appreciation of spirituality-based changes in the Innate Ring’s belief systems; autonomy-centred aspects within the Individual Ring; relational ties and societal expectations in the Relational and Societal Rings [20] respectively will help shepherd identity formation.

### The Krishna-Pisupati model

**Fig. 2:**
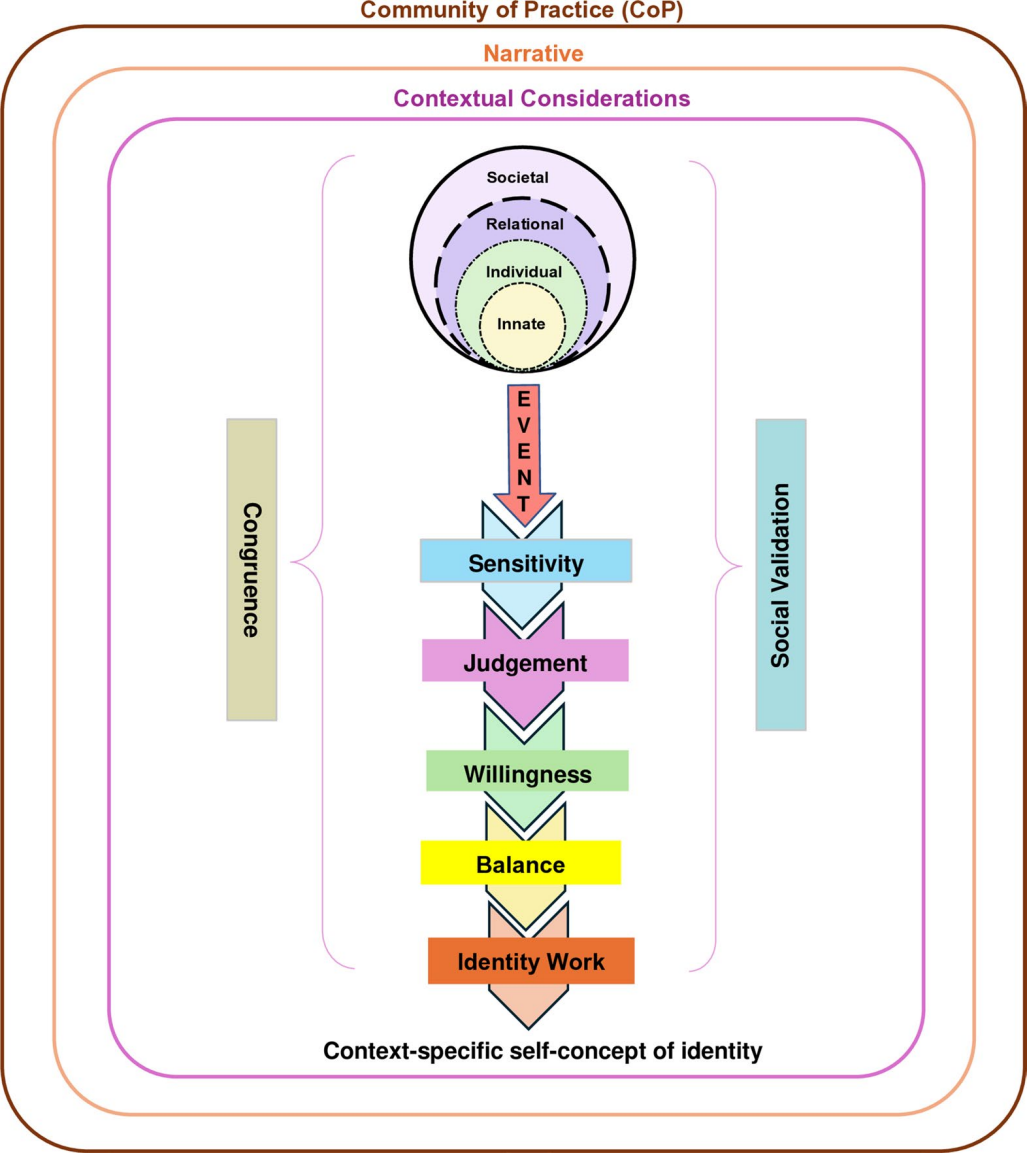
The Krishna-Pisupati Model for Professional Identity Formation

Changes in the belief systems are brought about by the introduction of new belief systems (*event*) that may resonate or conflict with regnant belief systems. Awareness of an *event* (*sensitivity*) precipitates *judgement* into the significance of the *event* and a determination as to the *willingness* to resolve the *event*. The balancing process considers the *willingness* and *judgement* of the significance of the *event* and weighs these against the sometimes-competing considerations of the stakeholder’s competence, experience and availability in the creation of a context-specific self-concept of identity as outlined in Fig. 2.

## Methods

**Fig. 3:**
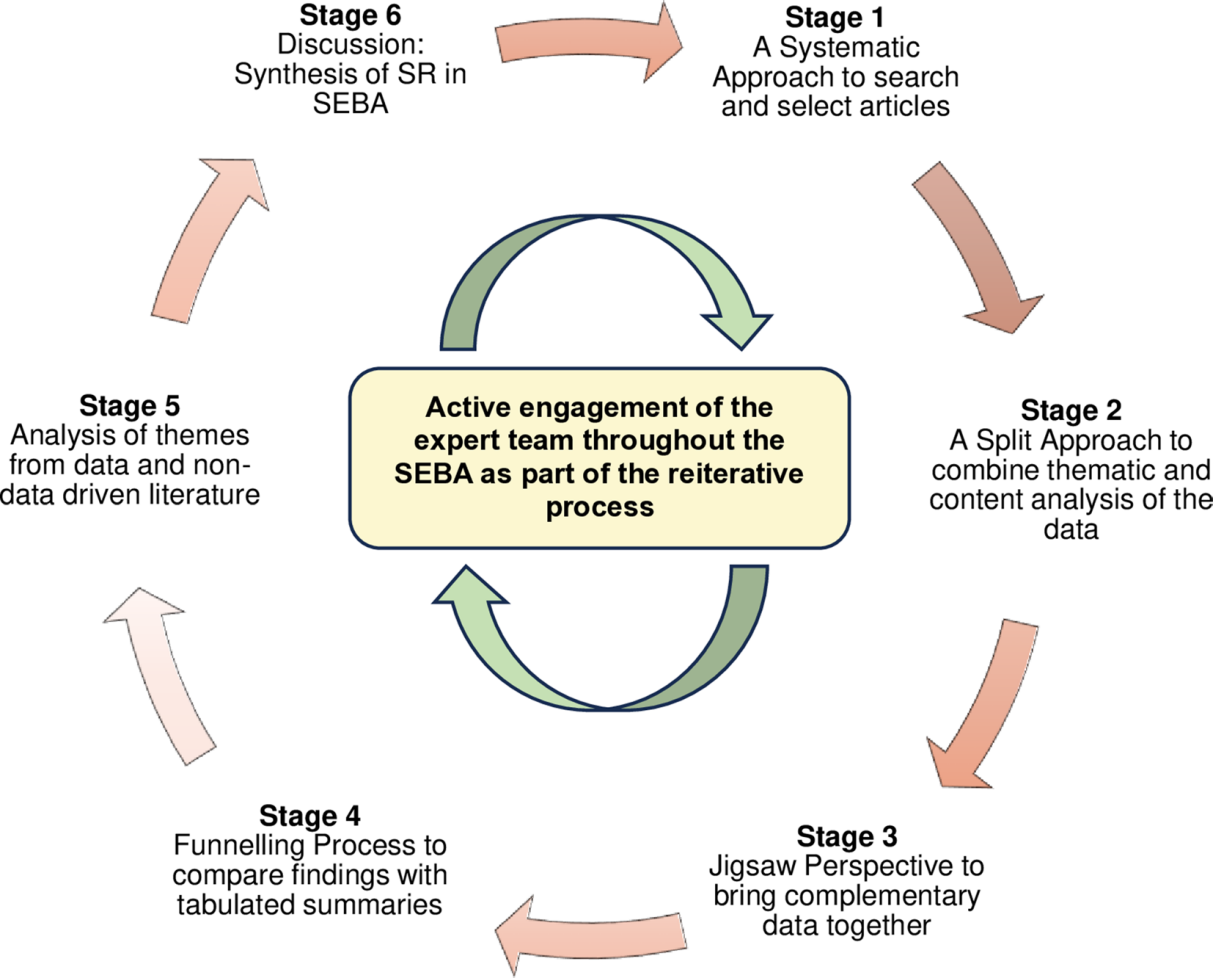
The SEBA Process

The PRISMA-compliant Systematic Evidence-Based Approach (SEBA) was adopted as the underlying methodologic framework for this systematic scoping review (see Additional File 1). Comprising six distinct stages, SEBA’s constructivist perspective and relativist lens accommodate the context-specific and socioculturally sensitive nature of mentoring, enabling a multi-angled mapping of existing literature for a consistent and reproducible review. The stages of SEBA are depicted in Fig. 3.

### Stage 1 of SEBA: A systematic approach

To enhance reproducibility and accountability of the research process, the stages of SEBA were guided and supported by an expert team [80, 81]. This comprised medical librarians, local educational experts and clinical practitioners from the Yong Loo Lin School of Medicine, the National Cancer Centre Singapore, the Palliative Care Institute Liverpool and Duke-NUS Medical School.

#### Determining the title, research Question(s) and inclusion criteria

The Population, Intervention, Comparison, Outcome, Study Design (PICOS) framework guided the primary research question, ‘*What is known about mentoring relationships in medical education and its impact on professional identity formation?*’ and secondary questions, ‘*How*,* in terms of the exact mechanisms*,* do mentoring relationships influence professional identity formation?*’ and ‘*What specific aspects of mentoring relationships influence professional identity formation?*’ (Table 2).

**Table 2:** Population, intervention, comparison, outcome and study design (PICOS) framework, inclusion and exclusion criteria applied to database search

PICOs	Inclusion	Exclusion
**Population**	Physicians, junior physicians, residents and medical students	Allied health specialties (e.g. nursing, psychology)
**Intervention**	Accounts of mentoring involving junior physicians, residents and/or medical students mentored by seniors aimed at advancing professional and/or personal development of the mentee with specific analysis on the role of the mentoring relationship	Peer-mentoring, mentoring patients, or mentoring by patients
**Comparison**	Comparisons between mentoring programmes, editorials and perspectives, reflective, narratives and opinion pieces	
**Outcome**	Personal outcomes of mentoring Professional development of outcomes Career-related outcomes Research and academia outcomes	
**Study design**	All study designs are included	

#### Searching

The research team conducted independent searches on PubMed, Embase, ERIC and Scopus databases. In trying to achieve an up-to-date review and given time and manpower constraints, we confined the search to articles published between 1st January 2000 and 4th December 2024. The research team opted to include studies published from the year 2000 onward to achieve balance between conducting a thorough review and ensuring that the included studies reflected current perspectives and practices in mentoring. To further enhance the review, ‘snowballing’ of references from the selected articles was performed, including through the use of artificial intelligence tools such as GPT and Elicit. An example of the search strategy is detailed in Table 3 below.

**Table 3:** Search strategy for pubmed database

**PubMed Search Strategy**
(“Mentors”[Mesh] OR mentor*[tiab]) AND (“Education, Medical” [Mesh] OR “Schools, Medical” [Mesh] OR “Students, Medical” [Mesh] OR “medical student*”[tiab] OR “medical school*”[tiab] OR “medical educat*”[tiab] OR “medical undergraduate*”[tiab] OR “medical postgraduate*”[tiab]) AND (“relation*” [tiab] OR “interaction*”[tiab] OR “dynamic*”[tiab] or “interpersonal”[tiab] or “inter-personal”[tiab] or “connection*”[tiab]) AND (“Professional identi*”[tiab] OR “Social Identification” [Mesh] OR “Socialization”[Mesh] OR “Social Identification” [tiab] OR “socialisation”[tiab] OR “socialization”[tiab])

#### Extracting and charting

Subsequently, the research team independently reviewed titles and abstracts using Endnote. This allowed for a shortlisting of articles, for which the full texts were reviewed. *“Negotiated consensual validation”* [82] was practiced to reach consensus on the final list of included articles. These list of articles are detailed in Additional File 2.

### Stage 2 of SEBA: split approach

Three independent teams then concurrently analysed the included full-text articles for a robust and comprehensive review. This involved the simultaneous application of Braun and Clarke’s [83] thematic analysis and Hsieh and Shannon’s [84] directed content analysis. The combined use of these approaches facilitated a shared understanding of terminology amongst various team members and addressed the limitations of each method of data analysis [85]. For example, contradictory data and negative findings often overlooked in thematic analyses are effectively accounted for in content analysis [83].

The first team summarised and tabulated the included articles, in keeping with Wong et al.’s [86] ‘Realist and Meta-narrative Evidence Syntheses - Evolving Standards (RAMESES) publication standards’ and Popay et al.’s [87] ‘Guidance on the conduct of narrative synthesis in systematic reviews’. This ensured that elemental details of the articles were captured.

The second team thematically analysed the articles, employing an inductive approach to construct ‘codes’ from the text’s immediate meaning [83]. This iterative process saw new codes linked to prior ones, ensuring that fresh themes were derived directly from the raw data without any pre-existing groupings [88].

The third team of researchers performed Hsieh and Shannon’s [84] directed content analysis, utilising pre-determined codes from Venktaramana et al.’s [70] review entitled, ‘Understanding mentoring relationships between mentees, peers and senior mentors’ and Sng et al.’s [89] review entitled, ‘Mentoring relationships between senior physicians and junior doctors and/or medical students: A thematic review’ to guide data analysis.

Similarly, *“negotiated consensual validation”* [82] was used to agree upon the end products of this triadic approach.

### Stages 3 and 4 of SEBA: Jigsaw perspective and funnelling process

Overlapping or complementary pieces were merged to create bigger puzzle pieces, referred to as themes/categories. These themes/categories were then compared with the tabulated summaries to ensure that key information was retained whilst minimising omissions. Overarching domains were subsequently identified.

### Stage 5 of SEBA: analysis of Evidence-Based and Non-Data-Driven literature

Efforts to minimise the plausibility of bias from non-data-based articles (grey literature, opinion, perspectives, editorial, letters) were seen in the comparisons made between evidenced-based and non-data-based publications. Found to yield similar data, the research team concluded that there was minimal bias from non-data-driven literature.

### Stage 6 of SEBA: synthesis of SSR in SEBA

Synthesis of this discussion waas guided by the Best Evidence Medical Education (BEME) Collaboration guide and the STORIES (STructured apprOach to the Reporting In healthcare education of Evidence Synthesis) statement [90, 91].

## Results

**Fig. 4:**
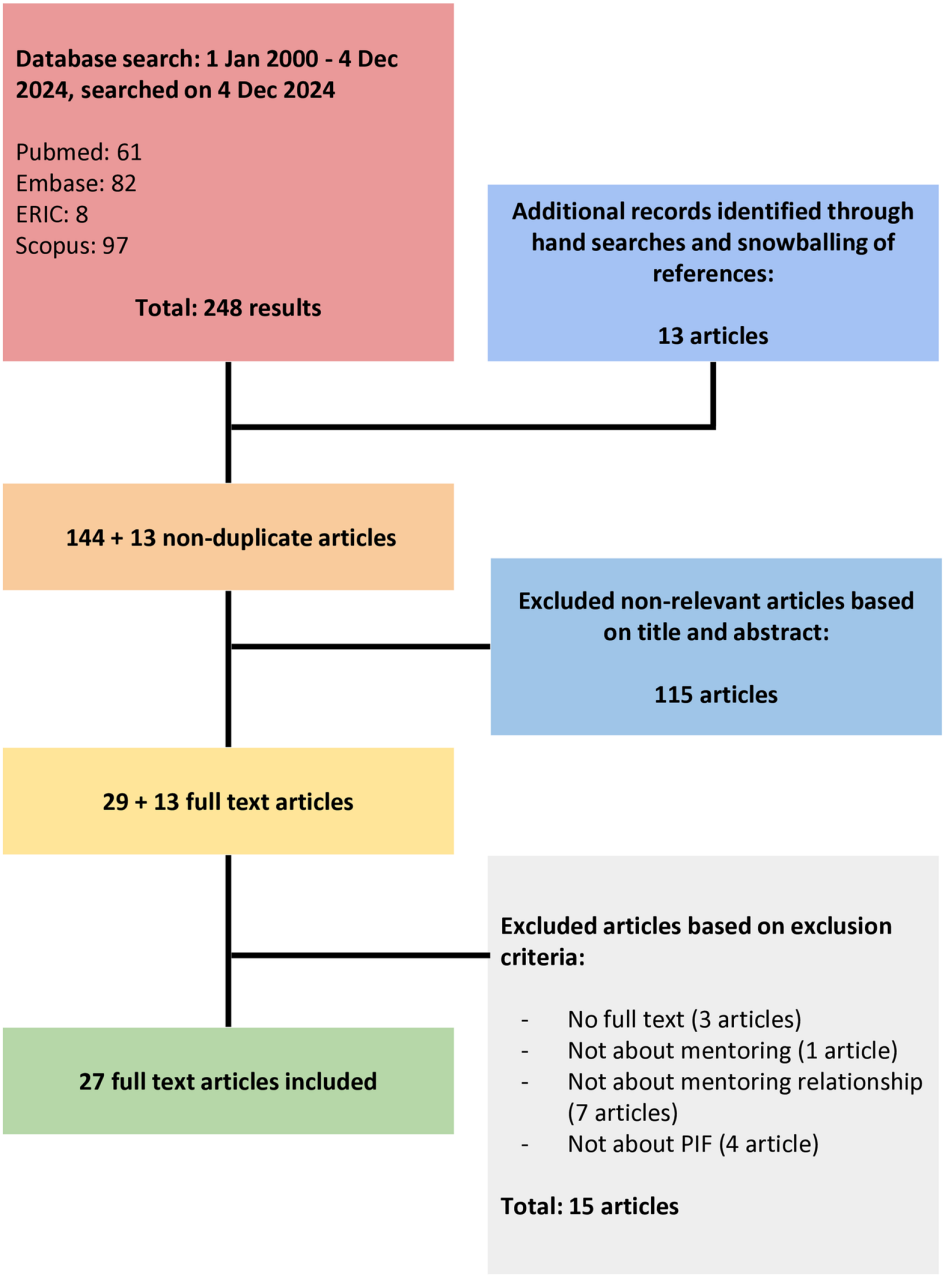
PRISMA Flowchart

248 articles were identified across four databases, with an additional 13 articles retrieved through hand-searches and snowballing of references. 157 non-duplicate titles and abstracts were reviewed, leading to 42 full-text reviews and a final inclusion of 27 articles (Fig. 4).

### Characteristics of included articles

Eight reviews, five commentaries, five quantitative studies and nine qualitative studies were included in this study. Amongst the five quantitative studies, Anurat et al. [77] utilised validated survey tools, such as Mentor Behaviour Scale, the Maslach Burnout Inventory Student Survey and the Professional Self -Identity Questionnaire. Chen et al. [92] utilised a usability survey regarding its novel mentorship mentoring platform. Heeneman and de Grave [69] designed and validated their mentorship experience tool for mentors and mentees whilst Kusner et al. [93] similarly designed a mentorship experience survey tool for mentors and mentees. Krishna et al. [8] designed content valid survey tools using a Modified Delphi approach with open-ended questions to understand mentoring experiences on the basis of mentoring stages. Qualitative interviews were carried out on both mentors [51, 74] and mentees [3, 5, 7, 70, 72, 75, 94] to understand their mentoring relationships and impact on professional identity formation. Uniquely, five of the interview studies involved a research-based mentoring programme with near-peer mentors and senior mentors [3, 5, 7, 70, 72].

The key domains identified were: (1) the mentoring ecosystem; (2) mentoring dynamics; (3) shifts in belief systems and professional identity; and (4) complex adaptive systems.

### Domain 1. The mentoring ecosystem

Personalised and enduring mentoring relationships are nurtured in mentoring ecosystems. A mentoring ecosystem is scaffolded on three key elements. One, the mentoring programme must function as a community of practice (CoP), or *“a persistent*,* sustaining social network of individuals who share and develop an overlapping knowledge base*,* set of beliefs*,* values and history and experiences focused on a common practice and/or enterprise”* [95]. This creates discrete boundaries made up of the programme’s inclusion and membership criteria, goals, compliance of mentoring standards, codes of practice, professional guidelines and expectations, ethical principles and medicolegal requirements [3, 5, 8, 51, 70, 72, 75–77, 93, 96–100]. These features also guide the mentoring trajectory [1, 3, 5, 6, 51, 69–72, 76, 97–100].

Two, the mentoring trajectory—framed by competency-based mentoring stages and supported by a longitudinal mentoring umbrella-based support mechanism and communication, assessment and feedback channels—shapes the mentoring ecosystem [1, 3, 6, 51, 70–72, 76, 97, 98, 100]. Progress along the mentoring trajectory maps achievement of stage-specific milestones [3, 5, 8, 70, 72, 76, 96, 97, 101].

Three, the mentoring ecosystem is also defined by its culture that is shaped by *individual*, *contextual*, *evolving* and *host organisational considerations*. The *individual considerations* refer to the stakeholder’s narratives, belief systems, psycho-emotional state, coping strategies and maturing mentoring relationship and competencies [1, 3, 5–7, 51, 69, 70, 72, 76, 98–101]. *Contextual considerations* include formal, informal and hidden curriculum; codes of conduct; access to personalised, appropriate and longitudinal mentoring support; assessment and remedial guidance [1, 3, 5, 8, 20, 69, 72, 75, 76, 92, 93, 96, 97, 99, 101]. The *evolving considerations* include growing personal and professional experience and competencies; maturing mentoring relationships; the cumulative effects of reflections and meaning-making exercises; deeper association and sense of belonging; and shifting belief systems [1, 3, 5–7, 12, 51, 70, 72, 74–76, 98–100]. The *host organisational considerations* encompass organisational support for mentoring assessments, mentor training and maintenance of the mentoring environment [6, 69, 93, 96–98].

### Domain 2. Mentoring dynamics

Within the confines of a well-structured and supported mentoring ecosystem, enduring and personalised mentoring relationships flourish [8, 69, 70, 72, 76, 96, 97, 100] as mentees are provided with guided immersion into the mentoring culture under the watchful eye of trained mentors [1, 5–7, 12, 51, 70–76, 98–101]. Guided by the mentoring trajectory and supported by role modelling, coaching, supervision, counselling and personalised mentoring support, trained faculty introduce and integrate *“the characteristics*,* values*,* and norms of the medical profession”* [102] through which mentees learn to effectively navigate the mentoring ecosystem by internalising its values and norms. The new belief systems and guidance provided sets expectations, guides interactions and shepherds the mentee from legitimate peripheral participation to more central roles in the programme as belief systems inculcated begin to take ‘root’, promoting investment in the mentoring relationship [5, 16, 73–75, 77].

### Domain 3. Shifts in belief systems and professional identity

Rooted belief systems inform the mentee’s personal and professional development [1, 3, 5–7, 51, 70, 72, 76, 98–100]. However, this process of professional identity formation is non-linear, evolving and adaptive—shaped by new experiences, challenges and the mentee’s ability to detect, evaluate and address conflicts between current and new belief systems [7, 98–100]. Mentors support this process by helping mentees develop their *internal compass*, or guiding values, beliefs and principles [1, 3, 5–7, 12, 51, 70, 72, 74–77, 98–101].

### Domain 4. Complex adaptive systems

Meaning-making, development of the *internal compass*, evolution of identity formation and creation of a context-dependent sense of identity are highly individualised and dependent upon organisational or environmental, stakeholder and relational facets of mentoring relationships.

Organisational factors include structured, multi-staged competency-based mentoring stages [3, 5, 8, 70, 72, 76, 96, 97, 101] that reveal changing expectations, milestones and shifts in thinking and competencies; the presence of an evolving but safe and nurturing mentoring environment in medicine’s hierarchical society [3, 8, 20, 75–77, 96, 97, 101]; formal and informal matching processes within the curricula [3, 8, 20, 70, 72, 76, 92, 93, 97]; resource variations; organisational and programme bureaucracy and access to trained faculty. These spotlight yet more variables to current interactions [12, 20, 96]. Krishna et al. [96], Anurat et al. [77] and Chen et al. [92] suggest that these variables have meaningful effects on the development of trusted mentoring interactions [20, 69, 72, 75, 76, 92].

Similarly, mentor-related factors, such as growing clinical knowledge; developing communication, debriefs, facilitation and leadership skills; maturing competencies [12, 69, 70, 72, 74–76]; shifts in personal and professional practice [69]; variations in motivation, engagement and availability as a source of social, professional and personal support [3, 5–7, 12, 20, 69–77, 101]; and the ability to build a sense of community [12, 69, 72, 76, 92, 93, 101], introduce yet more unpredictability in the mentoring relationship [5, 69, 72, 75, 76, 92].

Mentee-related factors, including developing trust, psychological safety [20, 69] and willingness to access available support, also underscore the complexities behind mentoring relationships.

Teo et al. [76] argue that the variable factors influencing identity work call into question current reliance on assessment tools built on *“Cartesian reductionism and Newtonian principles of linearity”*. In truth, such a linear cause-and-effect model is fundamentally flawed and fails to consider the unique influences, personalised responses and complex adaptations at play within a multi-stakeholder mentoring relationship; the effects of feedback loops; and the stakeholder’s psycho-emotional and contextual shifts, variations, availability of mentoring support and overall engagement in the mentoring process. Teo et al. [76] suggest that such complex interactions may be better understood through the lens of a complex adaptive system (CAS) [7, 76]—the *“dynamic interactions and variable interrelationships between and among their components that reverberate throughout the system”*. Indeed, Teo et al. [76] evidence the presence of defining characteristics of CAS in mentoring relationships, adding weight to their calls to change the manner that mentoring relationships are seen, understood and supported.

## Dicussion

In addressing its primary research question, this SEBA-guided review provides a unique perspective of developing mentoring relationships within a CoP-like mentoring ecosystem. Offering clear boundaries, a spiralled curriculum that guides a mentee from legitimate peripheral participation to a more central role and a mentoring culture that nurtures and supports the mentoring trajectory, a mentoring ecosystem scaffolds the socialisation process—providing the two essential elements in the development of PIF.

**Fig. 5:**
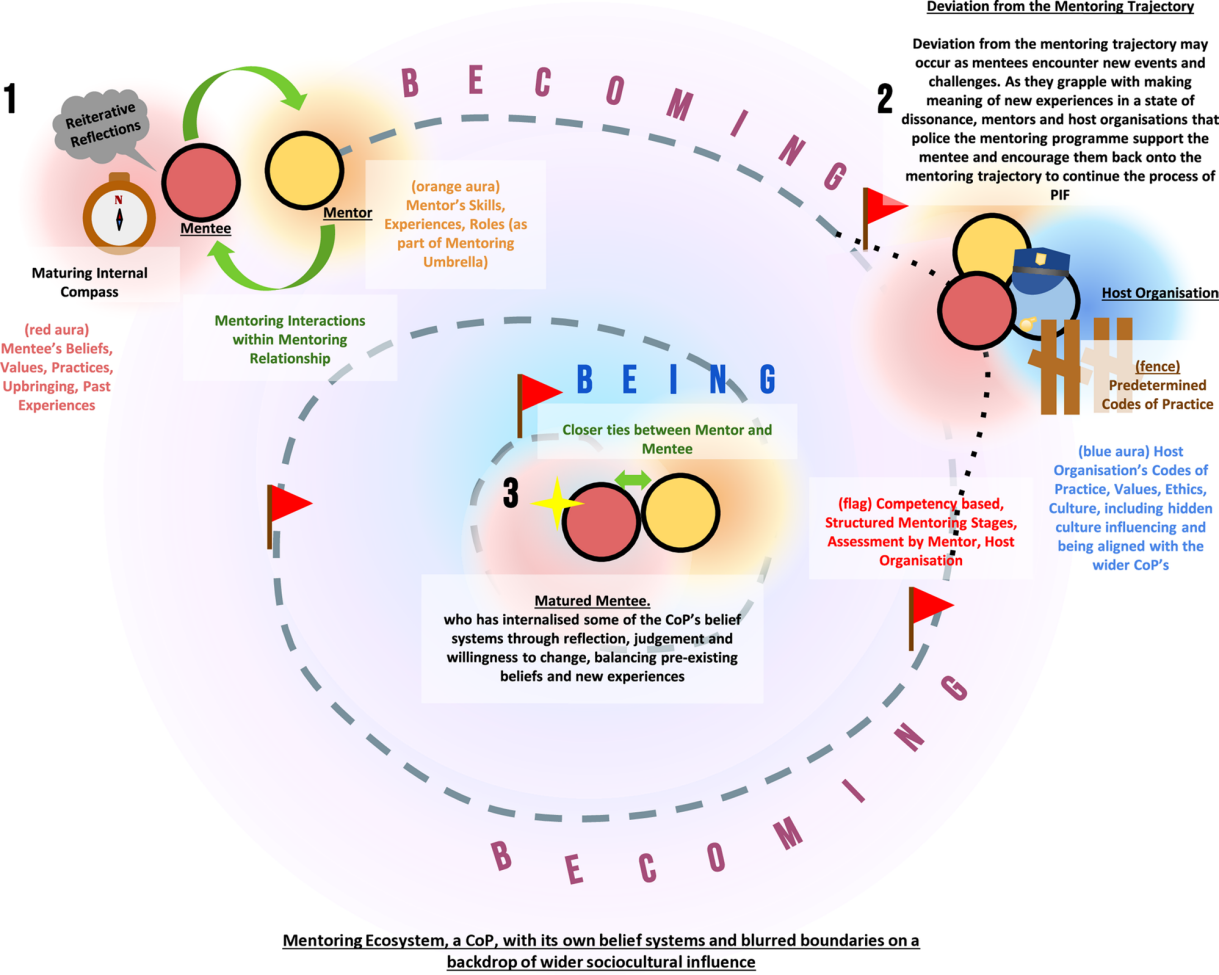
Navigating the Mentoring Trajectory on the Background of a Wider Mentoring Ecosystem

This SEBA-guided review proceeds to strengthen the role of mentoring relationship in scaffolding a mentee’s identity formation and professional development by underlining the importance of effective matching and an alignment of expectations in initiating mentoring relationships. Supported by guided immersion and longitudinal role modelling, supervision, coaching and counselling and personalised mentoring, the mentoring ecosystem, replete with its unique culture, builds individualised mentoring relationships. This longitudinal mentoring support paralleling the mentoring trajectory sustains engagement and encourages investment in the programme and instils common belief systems. As the programme’s shared identity takes root and becomes a part of the stakeholder’s core identity, these rooted belief systems and shared identity increase identification with the programme and inspires greater investment in the programme—allowing enduring and personalised mentoring relationships to blossom (Fig. 5).

This is part of the notion of *becoming*, where progress through the stages of the mentoring process, in tandem with guided reflections and discussions, supervised debriefs, timely feedback, and mentored meaning making, embed the programme’s belief systems and identity.

This building of the *internal compass* is critical to guiding thinking, decisioning and actions in resolving *events* or *dissonance* between new and prevailing belief systems and practices. The rooted belief systems and shared identity underpinning the *internal compass* increase identification with the programme and inspires even greater investment in resolving *events* and nurturing personalised and enduring mentoring relationships. Indeed, during an *event* (Point 2 in Fig. 5), a combination of the *internal compass* and policing regnant codes of conduct, professional guidelines and psychosocial expectations by the host organisation and the mentor helps identify the *dissonance* between new and prevailing belief systems—guiding the mentee as they review, reflect and make sense of their experiences and plan and execute a response. In some instances, the host organisation and mentors may be required to remediate these responses and shepherd the deviating mentoring relationship back towards the desired trajectory.

Over time, along the mentoring relationship, the *internal compass* matures (Point 3 in Fig. 5). This maturing notion of the *internal compass* remains relatively constant, even in the face of more changing conditions and different influences. Greater insights, feedback, reflections, reviews, experience, competency and experience add more layers of nuance to thinking, decisioning and conduct—underscoring a fledging sense of professional identity. It also accompanies a shift from legitimate peripheral participation to more central roles. This notion of a maturing *internal compass* alludes to another key finding. Sarraf-Yazdi et al. [2] posit that PIF is a continuous process of finessing and growing. Adding to this sense of *becoming* is also the practical notion of *being* an expert or, in this case, a senior mentor. However, rather than a fixed state of *being*, the role of senior mentor evolves. A senior mentor and local expert may have to take on the role of a relative novice in different settings. This demands the senior mentor strive to re-achieve an expert status. Changeability between expert and novice or some roles in between creates the sense of *being and becoming*. The concept of *being and becoming* highlights an evolving concept of professional identity that dismisses professional identity as a destination. It also reiterates the notion that professional development is a lifelong process.

Evidence of vacillations between *being and becoming*, evolving notions of personhood and identity and a maturing *internal compass* underscore the presence of a complex process. Evidence of multiple stakeholders interacting, adapting, co-evolving or mutually transforming in the face of changing circumstances, growing experience, shifting and e*volving individual*, *contextual* and *host organisational considerations* underscores evidence of mentoring relationships as a CAS.

Viewing mentoring relationships as CAS helps explain the impact of the rootedness of shared belief systems, gradual integration of shared identity and the development of enduring and personalised mentoring relationships. System adaptation or the ability to modify itself to maintain stability, optimise performance, or achieve objectives in light of practice changes, developing competencies, greater insights and nous, feedback loops and changing conditions also evidence features of self-organisation and emergent behaviour that extend beyond path dependency or the effects of past experiences and training. These features reflect more than the presence of a CAS, encapsulating the critical role of continued engagement and investment in the mentoring relationships that is elemental to creating enduring and personalised mentoring relationships. This is fundamental to the success of mentoring in the face of various influential factors swaying the progress of the mentoring relationship.

### Implications for practice

Our findings position mentorship as a powerful enabler of PIF in medical education, offering several practical implications for the design and implementation of effective mentorship programmes. To meaningfully support PIF, mentorship must embrace a holistic, personalised and longitudinal approach that encompasses the mentee’s evolving personal and professional identities.

Mentorship is found to be most impactful when it affirms competency, aligns expectations and fosters psychologically safe spaces for reflection and vulnerability. These elements can be actively cultivated through mentor training, structured check-ins and the development of clear communication frameworks. Moreover, as personal and professional identities are inseparably entwined, mentorship programmes should be intentionally designed to support the whole person. This is, after all, mentoring’s biggest strength—its unique ability to extend beyond formal training and provide personal support, serving as a developmental anchor throughout one’s career. Mentorship programmes should therefore build in continuity beyond training milestones, integrating mentorship into continuing medical education and career development pathways. Whilst it is clear further studies are warranted, mentoring has shown particular promise in delivering longitudinal, personalised support that bridges formal training and lifelong professional growth.

### Limitations

Small sample sizes, limitations posed by an inclusion criterion focused on publications in English and the use of predominantly Western-based data limit the applicability of these findings to other settings and healthcare and educational models.

## Conclusion

The implications of a maturing *internal compass* and sense of *being and becoming* are far-reaching. To begin, these concepts underscore the need for effective policing and support of programme boundaries; importance of consistency in the mentoring programme’s structure, practice, identity and shared belief systems; necessity for a curated mentoring programme and culture; demand for continued stakeholder engagement; and significance of effective assessments. The data also underlines the CoP-like structures, and guided immersion into the structured programme. This, in turn, underscores the necessary resources for longitudinal and personalised mentoring support.

Overall, the proffering of these insights underlines the need for further study which ought to begin with longitudinal studies and evaluation of the changes in PIF through the lens of the RToP and the KPM. This then will be the focus of our coming work.

## Electronic supplementary material

Below is the link to the electronic supplementary material.

Supplementary Material 1: PRISMA-ScR Checklist

Supplementary Material 2: Tabulated Summary of Included Articles

## Data Availability

The datasets supporting the conclusions of this article are included within the article and its additional files.

## Abbreviations

PIF: Professional Identity Formation
RToP: Ring Theory of Personhood
KPM: Krishna-Pisupati Model for Professional Identity Formation
SEBA: Systematic Evidence-Based Approach
PICOS: Population, Intervention, Comparison, Outcome, Study Design
CoP: Community of Practice
CAS: Complex Adaptive Systems

## References

[1] Cruess RL, Cruess SR, Boudreau JD, Snell L, Steinert Y. A schematic representation of the professional identity formation and socialization of medical students and residents: A guide for medical educators. Acad Med. 2015;90(6):718–25. 25785682 10.1097/ACM.0000000000000700

[2] Sarraf-Yazdi S, Goh S, Krishna L. Conceptualizing professional identity formation in medicine. Acad Med. 2023;99(3):343. 38015999 10.1097/ACM.0000000000005559

[3] Krishna LKR, Pisupati A, Ong YT, Teo KJH, Teo MYK, Venktaramana V, et al. Assessing the effects of a mentoring program on professional identity formation. BMC Med Educ. 2023;23(1):799. 37880728 10.1186/s12909-023-04748-6 PMC10601320

[4] Krishna LR. Towards an evidence based approach to novice mentoring in academic clinical practice: Lessons from internal medicine and practical applications for palliative medicine [thesis]. 2022.

[5] Krishna LKR, Pisupati A, Teo KJH, Teo MYK, Quek CWN, Chua KZY, et al. Professional identity formation amongst peer-mentors in a research-based mentoring programme. BMC Med Educ. 2023;23(1):787. 37875886 10.1186/s12909-023-04718-y PMC10598986

[6] Toh RQE, Koh KK, Lua JK, Wong RSM, Quah ELY, Panda A, et al. The role of mentoring, supervision, coaching, teaching and instruction on professional identity formation: A systematic scoping review. BMC Med Educ. 2022;22(1):531. 35804340 10.1186/s12909-022-03589-z PMC9270794

[7] Krishna LKR, Hamid NABA, Phua GLG, Mason S, Hill R, Lim C, et al. Peer mentorship and professional identity formation: an ecological systems perspective. BMC Med Educ. 2024;24(1):1007. 39278932 10.1186/s12909-024-05992-0 PMC11403841

[8] Krishna L, Tay KT, Yap HW, Koh ZYK, Ng YX, Ong YT, et al. Combined novice, near-peer, e-mentoring palliative medicine program: A mixed method study in Singapore. PLoS ONE. 2020;15(6):e0234322. 32502180 10.1371/journal.pone.0234322 PMC7274408

[9] Jingting W, Wahab M, Ikbal M, Wesley L, Kanesvaran R, Krishna L. Toward an interprofessional mentoring program in palliative care - a review of undergraduate and postgraduate mentoring in medicine, nursing, surgery and social work. J Palliat Care Med. 2016;6(6):1000292.

[10] Qiao Ting Low C, Toh YL, Teo SWA, Toh YP, Krishna L. A narrative review of mentoring programmes in general practice. Educ Prim Care. 2018;29(5):259–67. 30059278 10.1080/14739879.2018.1474723

[11] Teo KJH, Teo MYK, Pisupati A, Ong RSR, Goh CK, Seah CHX, et al. Assessing professional identity formation (PIF) amongst medical students in oncology and palliative medicine postings: A SEBA guided scoping review. BMC Palliat Care. 2022;21(1):200. 36397067 10.1186/s12904-022-01090-4 PMC9673314

[12] Koh EYH, Koh KK, Renganathan Y, Krishna L. Role modelling in professional identity formation: A systematic scoping review. BMC Med Educ. 2023;23(1):194. 36991373 10.1186/s12909-023-04144-0 PMC10052869

[13] Ong YT, Quek CWN, Pisupati A, Loh EKY, Venktaramana V, Chiam M, et al. Mentoring future mentors in undergraduate medical education. PLoS ONE. 2022;17(9):e0273358. 36108091 10.1371/journal.pone.0273358 PMC9477267

[14] Crampton PES, Afzali Y. Professional identity formation, intersectionality and equity in medical education. Med Educ. 2021;55(2):140–2. 33179338 10.1111/medu.14415

[15] Wyatt TR, Balmer D, Rockich-Winston N, Chow CJ, Richards J, Zaidi Z. Whispers and shadows’: A critical review of the professional identity literature with respect to minority physicians. Med Educ. 2021;55(2):148–58. 33448459 10.1111/medu.14295

[16] Chow CJ, Byington CL, Olson LM, Ramirez KPG, Zeng S, López AM. A conceptual model for Understanding academic physicians’ performances of identity: findings from the university of Utah. Acad Med. 2018;93(10):1539–49. 29794525 10.1097/ACM.0000000000002298 PMC6156991

[17] Ong E, Krishna L. Perspective from Singapore. Asian Bieoth Rev. 2014;6(4):420–7.

[18] Ong E, Krishna L, Neo P. The Sociocultural and ethical issues behind the decision for artificial hydration in a young palliative patient with recurrent intestinal obstruction. Ethics Med. 2015;31(1):39–51.

[19] Surbone A, Baider L. Personal values and cultural diversity. J Med Pers. 2013;11(1):11–8.

[20] Sarraf-Yazdi S, Teo YN, How AEH, Teo YH, Goh S, Kow CS, et al. A scoping review of professional identity formation in undergraduate medical education. J Gen Intern Med. 2021;36(11):3511–21. 34406582 10.1007/s11606-021-07024-9 PMC8606368

[21] Coetzee SK, Klopper HC. Compassion fatigue within nursing practice: A concept analysis. Nurs Health Sci. 2010;12(2):235–43. 20602697 10.1111/j.1442-2018.2010.00526.x

[22] Tedeschi RG, Calhoun LG. The posttraumatic growth inventory: measuring the positive legacy of trauma. J Trauma Stress. 1996;9(3):455–71. 8827649 10.1007/BF02103658

[23] McCann IL, Pearlman LA. Constructivist self-development theory: A theoretical framework for assessing and treating traumatized college students. J Am Coll Health. 1992;40(4):189–96. 1583241 10.1080/07448481.1992.9936281

[24] Cope A, Bezemer J, Mavroveli S, Kneebone R. What attitudes and values are incorporated into self as part of professional identity construction when becoming a surgeon? Acad Med. 2017;92(4):544–9. 28351068 10.1097/ACM.0000000000001454

[25] Au A. Online physicians, offline patients. Int J Sociol Soc Policy. 2018;38(5–6):474–83.

[26] Al-Abdulrazzaq D, Al-Fadhli A, Arshad A. Advanced medical students’ experiences and views on professionalism at Kuwait university. BMC Med Educ. 2014;14(1):150. 25056201 10.1186/1472-6920-14-150 PMC4118198

[27] Byszewski A, Hendelman W, McGuinty C, Moineau G. Wanted: role models - medical students’ perceptions of professionalism. BMC Med Educ. 2012;12(1):115. 23153359 10.1186/1472-6920-12-115 PMC3537482

[28] Kavas MV, Demirören M, Koşan AMA, Karahan ST, Yalim NY. Turkish students’ perceptions of professionalism at the beginning and at the end of medical education: A cross-sectional qualitative study. Med Educ Online. 2015;20(1):26614. 25795382 10.3402/meo.v20.26614 PMC4368711

[29] Smith SE, Tallentire VR, Cameron HS, Wood SM. The effects of contributing to patient care on medical students’ workplace learning. Med Educ. 2013;47(12):1184–96. 24206152 10.1111/medu.12217

[30] Kenny NP, Mann KV, MacLeod H. Role modeling in physicians’ professional formation: reconsidering an essential but untapped educational strategy. Acad Med. 2003;78(12):1203–10. 14660418 10.1097/00001888-200312000-00002

[31] Rosenblum ND, Kluijtmans M, ten Cate O. Professional identity formation and the clinician–scientist: A paradigm for a clinical career combining two distinct disciplines. Acad Med. 2016;91(12):1612–7. 27254011 10.1097/ACM.0000000000001252

[32] Meyer EM, Zapatka S, Brienza RS. The development of professional identity and the formation of teams in the veterans affairs Connecticut healthcare system’s center of excellence in primary care education program (CoEPCE). Acad Med. 2015;90(6):802–9. 25551857 10.1097/ACM.0000000000000594

[33] Birden H, Glass N, Wilson I, Harrison M, Usherwood T, Nass D. Teaching professionalism in medical education: A best evidence medical education (BEME) systematic review. BEME Guide 25 Med Teach. 2013;35(7):e1252–1266. 23829342 10.3109/0142159X.2013.789132

[34] Gilligan C, Loda T, Junne F, Zipfel S, Kelly B, Horton G, et al. Medical identity; perspectives of students from two countries. BMC Med Educ. 2020;20(1):420. 33172441 10.1186/s12909-020-02351-7 PMC7654572

[35] Wang XM, Swinton M, You JJ. Medical students’ experiences with goals of care discussions and their impact on professional identity formation. Med Educ. 2019;53(12):1230–42. 31750573 10.1111/medu.14006

[36] Witman Y. What do we transfer in case discussions? The hidden curriculum in medicine…. Perspect Med Educ. 2014;3(3):113–23. 24366760 10.1007/s40037-013-0101-0 PMC3976482

[37] Hafferty FW, Franks R. The hidden curriculum, ethics teaching, and the structure of medical education. Acad Med. 1994;69(11):861–71. 7945681 10.1097/00001888-199411000-00001

[38] Whitehead C, Kuper A, Freeman R, Grundland B, Webster F. Compassionate care? A critical discourse analysis of accreditation standards. Med Educ. 2014;48(6):632–43. 24807439 10.1111/medu.12429

[39] Seoane L, Tompkins LM, De Conciliis A, Boysen PG. 2nd. Virtues education in medical school: the foundation for professional formation. Ochsner J. 2016;16(1):50–5. 27046405 PMC4795502

[40] Gaufberg E, Bor D, Dinardo P, Krupat E, Pine E, Ogur B, et al. In pursuit of educational integrity: professional identity formation in the Harvard medical school Cambridge integrated clerkship. Perspect Biol Med. 2017;60(2):258–74. 29176087 10.1353/pbm.2017.0032

[41] Monrouxe LV. Identity, identification and medical education: why should we care? Med Educ. 2010;44(1):40–9. 20078755 10.1111/j.1365-2923.2009.03440.x

[42] Chuang AW, Nuthalapaty FS, Casey PM, Kaczmarczyk JM, Cullimore AJ, Dalrymple JL, et al. To the point: reviews in medical education - taking control of the hidden curriculum. Am J Obstet Gynecol. 2010;203(4):e316311–316. 10.1016/j.ajog.2010.04.035 20541735

[43] Kay D, Berry A, Coles NA. What experiences in medical school trigger professional identity development? Teach Learn Med. 2019;31(1):17–25. 29608109 10.1080/10401334.2018.1444487

[44] Jarvis-Selinger S, Pratt DD, Regehr G. Competency is not enough: integrating identity formation into the medical education discourse. Acad Med. 2012;87(9):1185–90. 22836834 10.1097/ACM.0b013e3182604968

[45] Frost HD, Regehr G. I am a Doctor: negotiating the discourses of standardization and diversity in professional identity construction. Acad Med. 2013;88(10):1570–7. 23969361 10.1097/ACM.0b013e3182a34b05

[46] Rodríguez C, López-Roig S, Pawlikowska T, Schweyer F-X, Bélanger E, Pastor-Mira MA, et al. The influence of academic discourses on medical students’ identification with the discipline of family medicine. Acad Med. 2015;90(5):660–70. 25406604 10.1097/ACM.0000000000000572

[47] MacLeod A. Caring, competence and professional identities in medical education. Adv Health Sci Educ Theory Pract. 2011;16(3):375–94. 21188513 10.1007/s10459-010-9269-9

[48] Warmington S, McColl G. Medical student stories of participation in patient care-related activities: the construction of relational identity. Adv Health Sci Educ Theory Pract. 2017;22(1):147–63. 27235124 10.1007/s10459-016-9689-2

[49] Foster K, Roberts C. The heroic and the villainous: A qualitative study characterising the role models that shaped senior Doctors’ professional identity. BMC Med Educ. 2016;16(1):206. 27530252 10.1186/s12909-016-0731-0 PMC4986406

[50] Hendelman W, Byszewski A. Formation of medical student professional identity: categorizing lapses of professionalism, and the learning environment. BMC Med Educ. 2014;14(1):139. 25004924 10.1186/1472-6920-14-139 PMC4102062

[51] Sternszus R, Boudreau JD, Cruess RL, Cruess SR, Macdonald ME, Steinert Y. Clinical teachers’ perceptions of their role in professional identity formation. Acad Med. 2020;95(10):1594–9. 32271232 10.1097/ACM.0000000000003369

[52] Jarvis-Selinger S, MacNeil KA, Costello GRL, Lee K, Holmes CL. Understanding professional identity formation in early clerkship: A novel framework. Acad Med. 2019;94(10):1574–80. 31192797 10.1097/ACM.0000000000002835

[53] Sadeghi Avval Shahr H, Yazdani S, Afshar L. Professional socialization: an analytical definition. J Med Ethics Hist Med. 2019;12:17. 32328230 10.18502/jmehm.v12i17.2016 PMC7166248

[54] Brody H, Doukas D, Professionalism. A framework to guide medical education. Med Educ. 2014;48(10):980–7. 10.1111/medu.12520 25200018

[55] Irby DM, Hamstra SJ. Parting the clouds: three professionalism frameworks in medical education. Acad Med. 2016;91(12):1606–11. 27119331 10.1097/ACM.0000000000001190

[56] Rosenthal S, Howard B, Schlussel YR, Herrigel D, Smolarz BG, Gable B, et al. Humanism at heart: preserving empathy in third-year medical students. Acad Med. 2011;86(3):350–8. 21248596 10.1097/ACM.0b013e318209897f

[57] Wright SM, Levine RB, Beasley B, Haidet P, Gress TW, Caccamese S, et al. Personal growth and its correlates during residency training. Med Educ. 2006;40(8):737–45. 16869918 10.1111/j.1365-2929.2006.02499.x

[58] Levine RB, Haidet P, Kern DE, Beasley BW, Bensinger L, Brady DW, et al. Personal growth during internship. J Gen Intern Med. 2006;21(6):564–9. 16808737 10.1111/j.1525-1497.2006.00383.x PMC1924625

[59] Fischer MA, Haley H-L, Saarinen CL, Chretien KC. Comparison of blogged and written reflections in two medicine clerkships. Med Educ. 2011;45(2):166–75. 21208262 10.1111/j.1365-2923.2010.03814.x

[60] Kern DE, Wright SM, Carrese JA, Lipkin M Jr., Simmons JM, Novack DH, et al. Personal growth in medical faculty: A qualitative study. West J Med. 2001;175(2):92–8. 11483549 10.1136/ewjm.175.2.92 PMC1071495

[61] Kimmons R, Veletsianos G. The fragmented educator 2.0: social networking sites, acceptable identity fragments, and the identity constellation. Comput Educ. 2014;72:292–301.

[62] Gosselink MJ. Medical weblogs: advocacy for positive cyber role models. Clin Teach. 2011;8(4):245–8. 22085000 10.1111/j.1743-498X.2011.00483.x

[63] Fieseler C, Meckel M, Ranzini G. Professional personae - how organizational identification shapes online identity in the workplace. J Comput Mediat Commun. 2014;20(2):153–70.

[64] Stokes J, Price B. Social media, visual culture and contemporary identity. Open Cybern Syst J. 2017;1:159–63

[65] Maghrabi RO, Oakley RL, Nemati HR. The impact of self-selected identity on productive or perverse social capital in social network sites. Comput Hum Behav. 2014;33:367–71.

[66] Hojat M, Vergare MJ, Maxwell K, Brainard G, Herrine SK, Isenberg GA, et al. The devil is in the third year: A longitudinal study of erosion of empathy in medical school. Acad Med. 2009;84(9):1182–91. 19707055 10.1097/ACM.0b013e3181b17e55

[67] Newton BW, Barber L, Clardy J, Cleveland E, O’Sullivan P. Is there hardening of the heart during medical school? Acad Med. 2008;83(3):244–9. 18316868 10.1097/ACM.0b013e3181637837

[68] Kaczmarczyk JM, Chuang A, Dugoff L, Abbott JF, Cullimore AJ, Dalrymple J, et al. E-professionalism: A new frontier in medical education. Teach Learn Med. 2013;25(2):165–70. 23530680 10.1080/10401334.2013.770741

[69] Heeneman S, de Grave W. Development and initial validation of a dual-purpose questionnaire capturing mentors’ and mentees’ perceptions and expectations of the mentoring process. BMC Med Educ. 2019;19(1):133. 31068162 10.1186/s12909-019-1574-2 PMC6505175

[70] Venktaramana V, Ong YT, Yeo JW, Pisupati A, Krishna LKR. Understanding mentoring relationships between mentees, peer and senior mentors. BMC Med Educ. 2023;23(1):76. 36717909 10.1186/s12909-023-04021-w PMC9887801

[71] Wald HS. Professional identity (trans)formation in medical education: reflection, relationship, resilience. Acad Med. 2015;90(6):701–6. 25881651 10.1097/ACM.0000000000000731

[72] Krishna L, Toh YP, Mason S, Kanesvaran R. Mentoring stages: A study of undergraduate mentoring in palliative medicine in Singapore. PLoS ONE. 2019;14(4):e0214643. 31017941 10.1371/journal.pone.0214643 PMC6481808

[73] Hee JM, Yap HW, Ong ZX, Quek SQM, Toh YP, Mason S, et al. Understanding the mentoring environment through thematic analysis of the learning environment in medical education: A systematic review. J Gen Intern Med. 2019;34(10):2190–9. 31011975 10.1007/s11606-019-05000-y PMC6816739

[74] Farrukh K, Mehr Y. Impact of mentor-mentee relationship on autonomy development of mentees in health professional education. J Fatima Jinnah Med Univ. 2023;17(1):3–6.

[75] Kalén S, Ponzer S, Silén C. The core of mentorship: medical students’ experiences of one-to-one mentoring in a clinical environment. Adv Health Sci Educ Theory Pract. 2012;17(3):389–401. 21792708 10.1007/s10459-011-9317-0

[76] Teo MYK, Ibrahim H, Lin CKR, Hamid NABA, Govindasamy R, Somasundaram N, et al. Mentoring as a complex adaptive system – a systematic scoping review of prevailing mentoring theories in medical education. BMC Med Educ. 2024;24(1):726. 38970020 10.1186/s12909-024-05707-5 PMC11225364

[77] Anurat K, Thamyongkit S, Pakakasama S, Sumrithe S. Assessing the role of mentors in mitigating burnout and enhancing professional development in medical education. Int J Med Educ. 2024;15:1–7. 38284420 10.5116/ijme.659b.d08c PMC11288619

[78] Radha Krishna LK, Alsuwaigh R. Understanding the fluid nature of personhood - the ring theory of personhood. Bioethics. 2015;29(3):171–81. 24547934 10.1111/bioe.12085

[79] Lim N-A, Ho C, Wong J, Peh R, Rahman N, Chiam M, et al. 2 Examining the ring theory of personhood (RToP) strategy: key components of dyssynchrony in end-of-life care. BMJ Support Palliat Care. 2023;13:A1.

[80] Liang JZ, Ng DKW, Raveendran V, Teo MYK, Quah ELY, Chua KZY, et al. The impact of online education during the covid-19 pandemic on the professional identity formation of medical students: A systematic scoping review. PLoS ONE. 2024;19(1):e0296367. 38181035 10.1371/journal.pone.0296367 PMC10769105

[81] Lim YX, Quah ELY, Chua KZY, Lin Ronggui CK, Govindasamy R, Ong SM, et al. A systematic scoping review on dignity assessment tools. J Pain Symptom Manage. 2024;67(4):e263–84. 38092260 10.1016/j.jpainsymman.2023.12.008

[82] Sandelowski M, Barroso J. Writing the proposal for a qualitative research methodology project. Qual Health Res. 2003;13(6):781–820. 12891715 10.1177/1049732303013006003

[83] Braun V, Clarke V. Using thematic analysis in psychology. Qual Res Psychol. 2006;3(2):77–101.

[84] Hsieh HF, Shannon SE. Three approaches to qualitative content analysis. Qual Health Res. 2005;15(9):1277–88. 16204405 10.1177/1049732305276687

[85] Burla N, Ong RSR, Chee RCH, Wong RSM, Neo SY, Abdul Hamid NAB, et al. A systematic scoping review on group non-written reflections in medical education. BMC Med Educ. 2024;24(1):1119. 39390436 10.1186/s12909-024-06117-3 PMC11468106

[86] Wong G, Greenhalgh T, Westhorp G, Buckingham J, Pawson R. RAMESES publication standards: Meta-narrative reviews. BMC Med. 2013;11(1):20. 23360661 10.1186/1741-7015-11-20 PMC3558334

[87] Popay J, Roberts H, Aj S, Petticrew M, Britten N, Arai L, et al. Guidance on the conduct of narrative synthesis in systematic reviews final report. J Epidemiol Community Health. 2005;59(Suppl 1):A7.

[88] Cassol H, Pétré B, Degrange S, Martial C, Charland-Verville V, Lallier F, et al. Qualitative thematic analysis of the phenomenology of near-death experiences. PLoS ONE. 2018;13(2):e0193001. 29444184 10.1371/journal.pone.0193001 PMC5812660

[89] Sng JH, Pei Y, Toh YP, Peh TY, Neo SH, Krishna LKR. Mentoring relationships between senior physicians and junior Doctors and/or medical students: A thematic review. Med Teach. 2017;39(8):866–75. 28562193 10.1080/0142159X.2017.1332360

[90] Haig A, Dozier M. BEME guide 3: systematic searching for evidence in medical education–part 2: constructing searches. Med Teach. 2003;25(5):463–84. 14522667 10.1080/01421590310001608667

[91] Frei E, Stamm M, Buddeberg-Fischer B. Mentoring programs for medical students-a review of the pubmed literature 2000–2008. BMC Med Educ. 2010;10(1):32. 20433727 10.1186/1472-6920-10-32 PMC2881011

[92] Chen JJ, Kusner JJ, Saldaña F, Potter J. Development of a novel mentorship platform to foster relational mentoring, empowered vulnerability, and professional identity formation in undergraduate medical education. Acad Med. 2021;96(11):1580–5. 33951683 10.1097/ACM.0000000000004152

[93] Kusner JJ, Chen JJ, Saldaña F, Potter J. Aligning student-faculty mentorship expectations and needs to promote professional identity formation in undergraduate medical education. J Med Educ Curric Dev. 2022;9:23821205221096307. 35572842 10.1177/23821205221096307 PMC9102129

[94] Chow J, Al-Duaij L, Last N, Sheth U, Rehman M, Azim A, et al. Transformational learning and professional identity formation in postgraduate competency-based medical education. Med Educ. 2025;59(4):409–17. 10.1111/medu.15553 PMC11906270 39440939

[95] Barab S, MaKinster J, Scheckler R. Designing system dualities: characterizing an online professional development. In: Barab S, Kling R, Gray J, editors. Designing for virtual communities in the service of learninges. Cambridge: Cambridge University Press; 2004. pp. 53–90.

[96] Krishna LKR, Renganathan Y, Tay KT, Tan BJX, Chong JY, Ching AH, et al. Educational roles as a continuum of mentoring’s role in medicine – a systematic review and thematic analysis of educational studies from 2000 to 2018. BMC Med Educ. 2019;19(1):439. 31775732 10.1186/s12909-019-1872-8 PMC6882248

[97] Krishna LKR, Tan LHE, Ong YT, Tay KT, Hee JM, Chiam M, et al. Enhancing mentoring in palliative care: an evidence based mentoring framework. J Med Educ Curric Dev. 2020;7:2382120520957649. 33015366 10.1177/2382120520957649 PMC7517982

[98] Cruess SR, Cruess RL, Steinert Y. Supporting the development of a professional identity: general principles. Med Teach. 2019;41(6):641–9. 30739517 10.1080/0142159X.2018.1536260

[99] Cruess RL, Cruess SR, Steinert Y. Medicine as a community of practice: implications for medical education. Acad Med. 2018;93(2):185–91. 28746073 10.1097/ACM.0000000000001826

[100] Sternszus R, Steinert Y, Razack S, Boudreau JD, Snell L, Cruess RL. Being, becoming, and belonging: reconceptualizing professional identity formation in medicine. Front Med (Lausanne). 2024;11:1438082. 39257893 10.3389/fmed.2024.1438082 PMC11383779

[101] Krishna LKR, Kwok HYF, Ravindran N, Tan XY, Soh J, Wan DWJ, et al. A systematic scoping review of mentoring support on professional identity formation. BMC Med Educ. 2024;24(1):1380. 39605048 10.1186/s12909-024-06357-3 PMC11600620

[102] Cruess RL, Cruess SR, Boudreau JD, Snell L, Steinert Y. Reframing medical education to support professional identity formation. Acad Med. 2014;89(11):1446–51. 25054423 10.1097/ACM.0000000000000427

