# Supplementary material for: The impact of mentoring relationships on professional identity formation in medical education: a systematic review
Source: BMC Med Educ. 2025 Apr 19;25:576. doi: 10.1186/s12909-025-07158-y (PMC12008893; doi:10.1186/s12909-025-07158-y)
Supplement: Supplementary file 2 — Supplementary Material 2: Tabulated Summary of Included Articles [file 12909_2025_7158_MOESM2_ESM.docx]

**Additional File 2. Summary of Included Articles**

| **SN** | **Title** | **Authors** | **Years** | **Study Type** | **MERSQI/COREQ** | **Introduction** | **Methods** | **Results** | **Conclusions** |
| --- | --- | --- | --- | --- | --- | --- | --- | --- | --- |
| **1** | Assessing the role of mentors in mitigating burnout and enhancing professional development in medical education | Anurat et al. | 2024 | Quantitative Survey | 12.5 | To assess the correlation between mentor behaviours and medical student burnout and their professional development within medical education. | A cross-sectional study using convenience sampling was conducted among third-, fifth-, and sixth-year medical students (N=307). Participants voluntarily completed anonymous online questionnaires measuring the Mentor Behavior Scale, the Maslach Burnout Inventory-Stu- dent Survey, and the Professional Self-Identity Questionnaire. Multivariate regression analysis was performed to analyse relationships between student burnout, mentor behaviours and their impact on professional development. | Among participants, 26% (N=80) experienced burnout, which was significantly associated with lower competency support (OR = 2.0, 95% CI: 1.1-3.5, p = 0.016), medication use (OR = 2.1, 95% CI: 1.1-4.0, p = 0.029), and a lower Grade Point Average (OR = 3.3, 95% CI: 1.6-6.9, p =0.001) compared to non-burnout students. In the develop- ment of professional identity, a high level of mentor relation- ship structure had statistically significant associations with higher scores in key domains of the Professional Self-Identity Questionnaire, including teamwork (OR = 3.9, 95% CI: 1.5- 9.9, p < 0.01), communication (OR = 3.4, 95% CI: 1.5-7.7, p < 0.01), ethical awareness (OR = 3.3, 95% CI: 1.4-8.0, p < 0.01), and record use (OR = 2.8, 95% CI: 1.2-6.5, p < 0.05). | The impact of mentor behaviours on medical students is evident. Enhancing mentorship by addressing specific mentor behaviours can improve programme quality. Future research should explore the long-term effects and strategies for effectively implementing targeted enhance- ments in mentor behaviours. |
| **2** | Development of a Novel Mentorship Platform to Foster Relational Mentoring, Empowered Vulnerability, and Professional Identity Formation in Undergraduate Medical Education | Chen et al | 2021 | Quantitative Survey | 7 | Mentorship is valuable to medical students undergoing professional identity formation. Many institutions lack infrastructure to facilitate the personalized mentoring that supports students’ integration of new professional identities with their personal identities and values. | The authors developed a novel mentorship platform called Weave via a multistep, iterative design process, incorporating in-person and survey- based student and faculty feedback. Features of Weave include clear communication of mentorship offerings and expectations, plus opportunities to engage mentors based on professional and personal (identity-based) attributes. Faculty at Harvard Medical School who created a mentor profile within the first 3 months of launch and students who visited the website within the same period were invited to complete usability surveys in February 2019; students were invited to complete impact surveys in August 2020. | Fifty-two of 132 invited faculty members (39.4%) and 80 of 185 students (43.2%) completed the usability surveys. Most of these faculty (86.5%) and students (73.8%) reported navigating the website was easy/very easy; 36 faculty (69.2%) created a mentor profile within 10 minutes. Key innovations highlighted by faculty and students were the listing of personal attributes and identities of diverse faculty; centralized, increased access to faculty mentors; ease of use; and provision of clear expectations.  Nearly all students who completed the impact surveys agreed that Weave allowed them to connect with a faculty mentor whom they would not have found through other sources and to learn about the dimensions of diverse faculty. | Weave is a customizable online mentorship platform that fosters empowered vulnerability and increases dialogue between medical students and faculty based on professional and personal interests and identities. Weave may be expanded to other mentoring contexts and adapted for implementation at other institutions to help cultivate an institutional culture that values mentoring and to strengthen broader diversity and inclusion efforts. |
| **3** | Impact of Mentor-Mentee Relationship on Autonomy development of Mentees in Health Professional Education | Farrukh et al | 2023 | Qualitative Interviews | 9 | There is growing evidence in literature on how student autonomy promotes motivational and well-being in medical students. Now a day there is lots of stress on medical students, mentoring is a simple technique to support our students but how mentors are to be train to give effective support is another challenge. Our study aims to explore the awareness of mentors on impact of mentor-mentee relation on autonomy development of mentees in health professional education. Mentor-mentee relationship can supports or constrain autonomy development of mentees this  study explored the phenomenon from mentor perspective. | Qualitative case study design and interpretivist paradigm was used; study duration was six months in bahria university health sciences campus. Data was collected using unstructured interviews until data was saturated. Four individual interviews of mentors using video eliciations, in which mentors reflected on their recent mentoring interaction with mentees and two focus group discussion of 90-120 minutes duration. Eight mentors were included in each focus group discussion. Total of twenty mentors participated in study by fulfilling inclusion creteria. Open-ended non-directive questions were asked. Self- determination theory was used for guiding interviews. Pattern matching was used for data analysis, it seeks to condense the par thematic units and synthesis of essential themes in order to describe them. | Essential themes were identified after data analysis, conversation on behaviour change, uncertainty on the extent to which they impact autonomy and social factors | Mentors were not certain on the extent to which they impact autonomy in mentees. Mentors training is required to develop skills of autonomy- supportiveness. Mentors should have clear guidelines regarding autonomy supportiveness well-being and professional growth. Autonomy supportiveness should continue parallel to professional identification development in undergraduate medical students |
| **4** | Development and initial validation of a dual-purpose questionnaire capturing mentors’ and mentees’ perceptions and expectations of the mentoring process | Heeneman et al | 2019 | Quantitative Survey | 10.5 | In health profession education, learners are often coached by mentors for development of competencies, self-direction of learning and professionalism. It is important that the mentee-mentor relationship is aligned in terms of mutual expectations. | A dual-purpose questionnaire capturing both the mentor and mentee perceptions on the actual and preferred mentoring functions was designed and validated, by performing a principal component analysis (PCA) using the data of mentees (n = 103) and mentors (n = 23) of a medical course. As a proof of concept, alignment of needs and changes in the mentoring perceptions in mentee groups of different years were determined. | PCA showed that specific sets of questions addressed important elements in the mentoring process, such as self-direction of learning and reflection (Scale 1), guidance of behavioural change (Scale 4), addressing personal issues and professional identity development (Scale 3 and 5) and how the mentor and mentee presents oneself in the mentoring relationship (Scale 2). Mentors and mentees perceived comparable situations as critical for an effective mentoring process, such as mentor presence and guidance of reflection, although there was also evidence of gaps, such as perception of cultural issues. By comparison of the mentee groups in the different years of the program, the dynamic or evolving nature of the mentor process became evident, mentees experienced more emphasis by the mentor on reflection (Scale 1), at a constant level of mentor presence (Scale 2). | Given the individualized, context-specific, and dynamic nature of mentoring, programmes would benefit from a regular evaluation of mentoring practices, e.g. by using questionnaires, in order to facilitate organizational revisions and further development of the mentoring competencies. |
| **5** | The core of mentorship: medical students’ experiences of one-to-one mentoring in a clinical environment | Kalen et al | 2012 | Qualitative Interviews | 14 | A mentoring program was set up where all medical students were offered a mentor during their first clinical courses; years 3–4. The mentors were physicians and their role as mentors was to support the students and act as sounding-boards, not to teach or assess knowledge. This study aimed to get a deeper understanding of the meaning of mentorship seen from the perspective of undergraduate medical students. A qualitative approach with individual interviews (N = 12) and inductive content analysis was chosen to investigate and interpret the meaning of mentorship | A mentoring program was set up where all medical students were offered a mentor during their first clinical courses; years 3–4. The mentors were physicians and their role as mentors was to support the students and act as sounding-boards, not to teach or assess knowledge. This study aimed to get a deeper understanding of the meaning of mentorship seen from the perspective of undergraduate medical students. A qualitative approach with individual interviews (N = 12) and inductive content analysis was chosen to investigate and interpret the meaning of mentorship | The results comprise three overarching themes: Space, Belief in the future and Transition. Having a mentor gave a sense of security and constituted a ‘free zone’ alongside the undergraduate programme. It gave hope about the future and increased motivation. The students were introduced to a new community and began to identify themselves as doctors. | We would argue that one-to-one mentoring can create conditions for medical students to start to develop some parts of the professional competences that are more elusive in medical education programmes, such as reflective capacity, emotional competence and the feeling of belonging to a community. |
| **6** | Assessing the effects of a mentoring program on professional identity formation | Krishna et al. | 2023 | Qualitative Interviews | 18 | Medical education has enjoyed mixed fortunes nurturing professional identity formation (PIF),  or how medical students think, feel and act as physicians. New data suggests that structured mentoring programs like the Palliative Medicine Initiative (PMI) may offer a means of developing PIF in a consistent manner. To better understand how a well-established structured research mentoring program shapes PIF, a study of the experiences of PMI mentees is proposed. | Acknowledging PIF as a sociocultural construct, a Constructivist approach and Relativist lens were adopted for this study. In the absence of an effective tool, the Ring Theory of Personhood (RToP) and Krishna-Pisupati Model (KPM) model were used to direct this dual Systematic Evidence-Based Approach (Dual-SEBA) study in design- ing, employing and analysing semi-structured interviews with PMI mentees and mentoring diaries. These served  to capture changes in PIF over the course of the PMI’s mentoring stages.  Transcripts of the interviews and mentoring diaries were concurrently analysed using content and thematic analy-  sis. Complementary themes and categories identified from the Split Approach were combined using the Jigsaw Approach and subsequently compared with mentoring diaries in the Funnelling Process. The domains created framed the discussion. | A total of 12 mentee interviews and 17 mentoring diaries were analysed, revealing two domains—PMI  as a Community of Practice (CoP) and Identity Formation. The domains confirmed the centrality of a structured CoP capable of facilitating longitudinal mentoring support and supporting the Socialisation Process along the mentoring trajectory whilst cultivating personalised and enduring mentoring relationships. | The provision of a consistent mentoring approach and personalised, longitudinal mentoring support guided along the mentoring trajectory by structured mentoring assessments lay the foundations for more effective mentoring programs. The onus must now be on developing assessment tools, such as a KPM-based tool, to guide support and oversight of mentoring relationships. |
| **7** | Aligning Student-Faculty Mentorship Expectations and Needs to Promote Professional Identity Formation in Undergraduate Medical Education | Kusner et al | 2022 | Quantitative Survey | 9 | During professional identity formation, medical students integrate their newly developing professional identities with their long- standing personal identities. Longitudinal mentorship has been shown to aid students in this process. Lack of clear relationship expectations among students and faculty is a barrier to effective longitudinal mentorship relationships. | A cross-sectional, survey-based study collected information about experiences from both students (mentees) and faculty (mentors). Surveys focused on collecting participants’ attitudes and expectations regarding ideal and actual mentorship experiences. Descriptive statistics and Fisher’s exact test analyses were used to compare the responses within and between students and faculty. | A total of 234 faculty and 181 medical students completed the survey. There were 187 faculty respondents (79.9%) who had previ- ously mentored students. Faculty who had versus had not previously mentored students differed significantly in their responses on the importance of mentors discussing aspects of their personal lives (71.1% vs. 54.3%, respectively, p = 0.0491), a quality valued by the majority of student respondents. As students progressed through medical school, they expressed increasing needs for personal mentorship and conversations regarding work/life integration and wellness (M1: 12.2%, M2: 18.8%, M3: 29.3%, M4: 51.7%). A minority of students (27% of M3 and 14.8% of M4 students) reported meeting faculty mentors through their clinical year experiences. | Faculty mentoring experience may improve student-faculty value alignment, which may in turn help to address student-identi- fied needs pertaining to personal development and professional identity formation in medical school. By contrasting student-identified mentorship expectations with those of faculty at various stages of mentorship experience, this study lays the groundwork for the formation of more effective longitudinal mentorship programs. |
| **8** | A Scoping Review of Professional Identity Formation in Undergraduate Medical Education | Sarraf-Yazdi et al | 2021 | Scoping Review | NIL | Professional identity formation (PIF) in medical students is a multifactorial phenomenon, shaped by ways that clinical and non-clinical experiences, expec- tations and environmental factors merge with individual values, beliefs and obligations. The relationship between students’ evolving professional identity and self-identity or personhood remains ill-defined, making it challenging for medical schools to support PIF systematically and strategically. Primarily, to capture prevailing literature on PIF in medical school education, and secondarily, to ascer- tain how PIF influences on medical students may be viewed through the lens of the ring theory of personhood (RToP) and to identify ways that medical schools support PIF. | A systematic scoping review was conducted using the systematic evidence-based approach. Articles published between 1 January 2000 and 1 July 2020 related to PIF in medical students were searched using PubMed, Embase, PsycINFO, ERIC and Scopus. Articles of all study designs (quantitative and qualitative), published or translat- ed into English, were included. Concurrent thematic and directed content analyses were used to evaluate the data. | A total of 10443 abstracts were identified, 272 full-text articles evaluated, and 76 articles included. The- matic and directed content analyses revealed similar themes and categories as follows: characteristics of PIF in relation to professionalism, role of socialization in PIF, PIF enablers and barriers, and medical school approaches to supporting PIF. | PIF involves iterative construction, decon- struction and inculcation of professional beliefs, values and behaviours into a pre-existent identity. Through the lens of RToP, factors were elucidated that promote or hin- der students’ identity development on individual relational or societal levels. If inadequately or inappropri- ately supported, enabling factors become barriers to PIF. Medical schools employ an all-encompassing approach to support PIF, illuminating the need for distinct and delib- erate longitudinal monitoring and mentoring to foster students’ balanced integration of personal and profes- sional identities over time. |
| **9** | Understanding mentoring relationships between mentees, peer and senior mentors | Venktaramana et al. | 2023 | Qualitative Interviews | 14 | Mentoring relationships play a critical but poorly understood role in mentoring’s overall success. To overcome these knowledge gaps, a study of mentee experiences in the Palliative Medicine Initiative, a structured research-based mentoring program, is proposed. The program’s clearly described mentoring approach, competency- based mentoring stages and curated mentoring environment ensure a consistent mentoring experience. It pro- vides a unique platform to study mentoring relationships longitudinally and its implications on professional identity formation. | The Tool Design Systematic Evidence-Based Approach methodology is used to map and employ current understanding. A review of recent reviews on mentoring processes, mentoring’s effects, professional identity formation and professional identity formation assessment tools lay the foundation for the design of semi-structured interviews and mentoring diaries to evaluate the characteristics of successful mentoring relationships and mentor- ing’s impact on professional identity formation. The data accrued from these tools were evaluated using this method- ology whilst changes in professional identity formation were assessed using the Ring Theory of Personhood | The semi-structured interviews revealed four themes: stakeholders, mentoring stages, mentoring relation- ships and professional identity formation whilst the mentoring diaries revealed two: mentoring processes and men- toring relationships. Two final domains emerged – mentoring relationships and professional identity formation. | The Palliative Medicine Initiative’s structured stage-based mentoring approach, trained stakeholders, curated environment, assessment-directed and personalized mentoring support reveal seven developmental stages of mentoring relationships. These culminate in changes to the values, beliefs and principles that shape how mentees see, feel and act as professionals. These findings suggest that mentoring programs may help to further develop and fine-tune their professional identity formation. |
| **10** | Professional Identity (Trans)Formation in Medical Education: Reflection, Relationship, Resilience | Wald et al | 2015 | Commentary | NIL | A fundamental goal of medical education is the active, constructive, transformative process of professional identity formation (PIF). Medical educators are thus  charged with designing standardized and personalized curricula for guiding, supporting, and challenging learners  on the developmental professional identity pathway, including the process of socialization. | NIL | NIL | Overarching lessons include attending to learners’ and faculty’s PIF within a developmental trajectory of the professional life cycle; process and content within PIF curricula as well as learners’ individual and collective voices; curricular/ extracurricular factors contributing to socialization, self-awareness, development of core values, and moral leadership; integrating PIF domains within pedagogy; faculty development for skilled mentoring and reflective coaching; and implementing resilience-promoting skill sets as “protective” within PIF. Outcomes assessment including the impact of curricula on learners and on patient- centered care can be challenging, and potential next steps toward this goal are discussed. |
| **11** | Mentoring stages: A study of undergraduate mentoring in palliative medicine in Singapore | Krishna et al. | 2018 | Qualitative Interviews | 15 | Mentoring nurtures a mentee’s personal and professional development. Yet conflation of mentoring approaches and a failure to contend with mentoring’s nature makes it difficult to study mentoring processes and relationships. This study aims to understand of mentee experiences in the Palliative Medicine Initiative (PMI). The PMI uses a consistent mentoring approach amongst ahomogeneous menteepopulation offers a unique opportunity to circumnavigate conflation of practices and the limitations posed by mentoring’s nature. The data will advance understanding of mentoring processes. | Sixteen mentees discussed their PMI experiences in individual face-to-face audio-recorded interviews. The two themes identified from thematic analysis of interview transcripts were the stages of mentoring and communication. | The6stagesof mentoring are the ‘pre-mentoring stage’, ‘initial research meetings’, ‘data gathering’, ‘review of initial findings, ‘manuscript preparation” and ‘reflections’. These subthemes sketch the progression of mentees from being dependent on the mentor for support andguidance, to an independent learner with capacity and willingness to mentor others. Eachsubthemeisdescribed as stages in the mentoring process (mentoring stages) given their association with a specific phase of the research process. Mentoring processes also pivot on effective communication which are influenced by the mentor’s characteristics and the nature of mentoring interactions. | Mentoring relationships evolve in stages to ensure particular competencies are met before mentees progress to the next part of their mentoring process. Progress is dependent upon effective communication and support from the mentor and appropriate and timely adaptations to the mentoring approach to meet the mentee’s needs and goals. Adaptations to the mentoring structure are informed by effective and holistic evaluation of the mentoring process and the mentor’s and mentee’s abilities, goals and situations. These findings underline the need to review and redesign the way assessments of the mentoring process are constructed and how mentoring programs are structured. |
| **12** | Educational roles as a continuum of mentoring’s role in medicine – a systematic review and thematic analysis of educational studies from 2000 to 2018 | Krishna et al | 2019 | Systematic Review | NIL | Recent studies have gone to great lengths to differentiate mentoring from teaching, tutoring, role modelling, coaching and supervision in efforts to better understand mentoring processes. This review seeks to evaluate the notion that teaching, tutoring, role modelling, coaching and supervision may in fact all be part of the mentoring process. To evaluate this theory, this review scrutinizes current literature on teaching, tutoring, role modelling, coaching and supervision to evaluate their commonalities with prevailing concepts of novice mentoring. | A three staged approach is adopted to evaluate this premise. Stage one involves four systematic reviews on one-to-one learning interactions in teaching, tutoring, role modelling, coaching and supervision within Internal Medicine, published between 1st January 2000 and 31st December 2018. Braun and Clarke’s (2006) approach to thematic analysis was used to identify key elements within these approaches and facilitate comparisons between them.  Stage two provides an updated view of one-to-one mentoring between a senior physician and a medical student or junior doctor to contextualise the discussion.  Stage three infuses mentoring into the findings delineated in stage one. | Seventeen thousand four hundred ninety-nine citations were reviewed, 235 full-text articles were reviewed, and 104 articles were thematically analysed. Four themes were identified – characteristics, processes, nature of relationship, and problems faced in each of the four educational roles. | Role modelling, teaching and tutoring, coaching and supervision lie within a mentoring spectrum of increasingly structured interactions, assisted by assessments, feedback and personalised support that culminate with a mentoring approach. Still requiring validation, these findings necessitate a reconceptualization of mentoring and changes to mentor training programs and how mentoring is assessed and supported. |
| **13** | Combined novice, near-peer, e-mentoring palliative medicine program: A mixed method study in Singapore | Krishna et al | 2020 | Quantitative  Survey | 6.5 | An acute shortage of senior mentors saw the Palliative Medicine Initiative (PMI) combine its novice mentoring program with electronic and peer mentoring to overcome insufficient mentoring support of medical students and junior doctors by senior clinicians. A three-phased evaluation was carried out to evaluate mentees’ experiences within the new CNEP mentoring program. | Phase 1 saw use of a Delphi process to create a content-valid questionnaire from data drawn from 9 systematic reviews of key aspects of novice mentoring. In Phase 2 Cognitive Interviews were used to evaluate the tool. The tool was then piloted amongst mentees in the CNEP program. Phase 3 compared mentee’s experiences in the CNEP program with those from the PMI’s novice mentoring program. | Thematic analysis of open-ended responses revealed three themes–the CNEP mentoring process, its benefits and challenges that expound on the descriptive statistical analysis of specific close-ended and Likert scale responses of the survey. The results show mentee experiences in the PMI’s novice mentoring program and the CNEP program to be similar and that the addition of near peer and e-mentoring processes enhance communications and support of mentees. | CNEP mentoring is an evolved form of novice mentoring built on a consistent mentoring approach supported by an effective host organization. The host organization marshals assessment, support and oversight of the program and allows flexibility within the approach to meet the particular needs of mentees, mentors and senior mentors. Whilst near-peer mentors and e-mentoring can make up for the lack of senior mentor availability, their effectiveness hinges upon a common mentoring approach.  To better support the CNEP program deeper understanding of the mentoring dynamics, policing and mentor and mentee training processes are required. The CNEP mentoring tool too needs to be validated. |
| **14** | Enhancing Mentoring in Palliative Care: An Evidence Based Mentoring Framework | Krishna et al | 2020 | Narrative Review | NIL | Growing concerns over ethical issues in mentoring in medicine and surgery have hindered efforts to reinitiate mentoring for Palliative Care (PC) physicians following the easing of COVID-19 restrictions. Ranging from the misappropriation of mentee’s work to bullying, ethical issues in mentoring are attributed to poor understanding and structuring of mentoring programs, underlining the need for a consistent approach to mentoring practices. | Given diverse practices across different settings and the employ of various methodologies, a novel approach to narrative reviews (NR)s is proposed to summarize, interpret, and critique prevailing data on novice mentoring. To overcome prevailing concerns surrounding the reproducibility and transparency of narrative reviews, the Systematic Evidenced Based Approach (SEBA) adopts a structured approach to searching and summarizing the included articles and employed concurrent content and thematic analysis that was overseen by a team of experts. | A total of 18 915 abstracts were reviewed, 62 full text articles evaluated and 41 articles included. Ten themes/categories were ascertained identified including Nature; Stakeholders; Relationship; Approach; Environment; Benefits; Barriers; Assessments; Theories and Definitions. | By compiling and scrutinizing prevailing practice it is possible to appreciate the notion of the mentoring ecosystem which sees each mentee, mentor, and host organization brings with them their own microenvironment that contains their respective goals, abilities, and contextual considerations. Built around competency based mentoring stages, it is possible to advance a flexible yet consistent novice mentoring framework. |
| **15** | Professional identity formation amongst peer-mentors in a research-based mentoring programme | Krishna et al | 2023 | Qualitative Interviews | 14 | Mentoring plays a pivotal yet poorly understood role in shaping a physician’s professional identity formation (PIF) or how they see, feel and act as professionals. New theories posit that mentoring nurtures PIF by functioning as a community of practice through its structured approach and its support of a socialisation process made possible by its assessment-directed personalized support. To test this theory and reshape the design, employ and support of mentoring programs, we evaluate peer-mentor experiences within the Palliative Medicine Initiative’s structured research mentoring program. | Semi-structured interviews with peer mentors under the Palliative Medicine Initiative (PMI) at National Cancer Centre Singapore were conducted and triangulated against mentoring diaries to capture longitudinal data of their PMI experiences. The Systematic Evidence-Based Approach (SEBA) was adopted to enhance the trustworthiness of the data. SEBA employed concurrent content and thematic analysis of the data to ensure a comprehensive review. The Jigsaw Perspective merged complementary themes and categories identified to create themes/categories. The themes/categories were compared with prevailing studies on mentoring in the Funnelling Process to reaffirm their accuracy. | Twelve peer-mentors participated in the interviews and eight peer-mentors completed the mentoring diaries. The domains identified were community of practice and identity work. | The PMI’s structured mentoring program functions as a community of practice supporting the socialisation process which shapes the peer-mentor’s belief system. Guided by a structured mentoring approach, stage-based assessments, and longitudinal mentoring and peer support, peer-mentors enhance their detection and evaluation of threats to their regnant belief system and adapt their self-concepts of identity and personhood to suit their context. These insights will help structure and support mentoring programs as they nurture PIF beyond Palliative Medicine. |
| **16** | Understanding the Mentoring Environment Through Thematic Analysis of the Learning Environment in Medical Education: a Systematic Review | Hee et al | 2018 | Systematic Review | NA | Mentoring’s success has been attributed to individualised matching, holistic mentoring relation- ships (MRs) and personalised mentoring environments (MEs). Whilst there is growing data on matching and MRs, a dearth of ME data has hindered development of mentoring programme. Inspired by studies likening MEs to learning environments (LEs) and data highlighting common characteristics between the two, this systematic review scrutinises reports on LEs to extrapolate the find- ings to the ME context to provide a better understanding of ME and their role in the mentoring process. | Using identical search strategies, 6 reviewers carried out independent literature reviews of LEs in clin- ical medicine published between 1 January 2000 and 31 December 2015 using PubMed, ERIC, Cochrane Data- base of Systematic Reviews, Google Scholar and Scopus databases. Braun and Clarke’s (2006) approach to the- matic analysis was adopted to circumnavigate LE’s evolv- ing, context-specific, goal-sensitive, learner-tutor rela- tionally dependent nature. | A total of 4574 abstracts were identified, 90 articles were reviewed, and 58 full-text articles were the- matically analysed. The two themes identified were LE structure and LE culture. LE structure regards the frame- work that guides interactions within the LE. LE culture concerns the values and practices influencing learner- tutor-host organisation interactions. | LE is the product of culture and structure that influence and are influenced by the tutor-learner-host organisation relationship. LE structure guides the evolving tutor-learner-host organisation relationship whilst the LE culture nurtures it and oversees the LE structure. Similar- ities between LEs and MEs allow LE data to inform programme designers of ME’s role in mentoring’s success. |
| **17** | The role of mentoring, supervision, coaching, teaching and instruction on professional identity formation: a systematic scoping review | Toh et al | 2022 | Systematic Review | NIL | Mentoring’s pivotal role in nurturing professional identity formation (PIF) owes much to its combined use with supervision, coaching, tutoring, instruction, and teaching. However the effects of this combination called the ‘mentoring umbrella’ remains poorly understood. This systematic scoping review thus aims to map current understanding. | A Systematic Evidence‑Based Approach guided systematic scoping review seeks to map current understanding of the ‘mentoring umbrella’ and its effects on PIF on medical students and physicians in training. It is hoped that insights provided will guide structuring, support and oversight of the ‘mentoring umbrella’ in nurturing PIF. Articles published between 2000 and 2021 in PubMed, Scopus, ERIC and the Cochrane databases were scrutinised. The included articles were concurrently summarised and tabulated and concurrently analysed using content and thematic analysis and tabulated. The themes and categories identified were compared with the summaries of the included articles to create accountable and reproducible domains that guide the discussion. | A total of 12201 abstracts were reviewed, 657 full text articles evaluated, and 207 articles included. The three domains identified were definitions; impact on PIF; and enablers and barriers. The mentoring umbrella shapes PIF in 3 stages and builds a cognitive base of essential knowledge, skills and professional attitudes. The cognitive base informs thinking, conduct and opinions in early supervised clinical exposure in Communities of practice (COP). The COPs’ individualised approach to the inculcation of desired professional characteristics, goals, values, principles and beliefs reshapes the individual’s identity whilst the socialisation process sees to their integration into current identities. | The mentoring umbrella’s provides personalised longitudinal support in the COP and socialisation process. Understanding it is key to addressing difficulties faced and ensuring holistic and timely support. |
| **18** | Mentoring as a complex adaptive system – a systematic scoping review of prevailing mentoring theories in medical education | Teo et al | 2024 | Systematic Review | NIL | Effective mentorship is an important component of medical education with benefits to all stakeholders. In recent years, conceptualization of mentorship has gone beyond the traditional dyadic experienced mentor-novice mentee relationship to include group and peer mentoring. Existing theories of mentorship do not recognize mentoring’s personalized, evolving, goal-driven, and context-specific nature. Evidencing the limitations of traditional cause-and-effect concepts, the purpose of this review was to systematically search the literature to determine if mentoring can be viewed as a complex adaptive system (CAS). | A systematic scoping review using Krishna’s Systematic Evidence-Based Approach was employed to study medical student and resident accounts of mentoring and CAS in general internal medicine and related subspecialties in articles published between 1 January 2000 and 31 December 2023 in PubMed, Embase, PsycINFO, ERIC, Google Scholar, and Scopus databases. The included articles underwent thematic and content analysis, with the themes identified and combined to create domains, which framed the discussion. | Of 5,704 abstracts reviewed, 134 full-text articles were evaluated, and 216 articles were included. The domains described how mentoring relationships and mentoring approaches embody characteristics of CAS and that mentorship often behaves as a community of practice (CoP). Mentoring’s CAS-like features are displayed through CoPs, with distinct boundaries, a spiral mentoring trajectory, and longitudinal mentoring support and assessment processes. | Recognizing mentorship as a CAS demands the rethinking of the design, support, assessment, and oversight of mentorship and the role of mentors. Further study is required to better assess the mentoring process and to provide optimal training and support to mentors. |
| **19** | Peer mentorship and professional identity formation: an ecological systems perspective | Krishna et al | 2024 | Qualitative Interview Study | 18 | Mentoring can help shape how medical students think, feel, and act as physicians. Yet, the mechanism in which it influences this process of professional identity formation (PIF) remains poorly understood. Through the lens of the ecological systems theory, this study explores the interconnected and dynamic system of mentoring relationships and resources that support professional development and growth within the Palliative Medicine Initiative (PMI), a structured research peer mentoring program. | A secondary analysis of transcripts of semi-structured interviews with peer mentors and mentees and a review of their mentoring diaries was conducted to explore the impact of participation in a longitudinal peer mentoring program on both mentees and peer mentors on their personal and professional development through the lens of the mentoring ecosystem model. The Systematic Evidence-Based Approach was adapted to analyze the data via content and thematic analysis. | Eighteen mentees and peer mentors participated and described a supportive community of practice within the research program, with discrete micro-, meso-, and macro-environments that are dynamic, reflexive, and interconnected to form a mentoring ecosystem. Within this ecosystem, reflection is fostered, and identity work is done—ultimately shaping and refining self-concepts of personhood and identity. | This study underscores the nuances and complexities of mentorship and supports the role of the mentoring ecosystem in PIF. A deeper understanding of the multiple factors that converge to facilitate the professional development of mentees can help educators develop and implement structured peer mentorship programs that better support reflective practice and identity work. |
| **20** | Supporting the development of a professional identity: General principles | Cruess et al | 2019 | Commentary | NIL | While teaching medical professionalism has been an important aspect of medical education over the past two decades, the recent emergence of professional identity formation as an important concept has led to a reexamination of how best to ensure that medical graduates come to “think, act, and feel like a physician.” If the recommendation that professional iden- tity formation as an educational objective becomes a reality, curricular change to support this objective is required and the principles that guided programs designed to teach professionalism must be reexamined. | NIL | NIL | It is proposed that the social learn- ing theory communities of practice serve as the theoretical basis of the curricular revision as the theory is strongly linked to identity formation. Curricular changes that support professional identity formation include: the necessity to establish identity formation as an educational objective, include a cognitive base on the subject in the formal curriculum, to engage students in the development of their own identities, provide a welcoming community that facilitates their entry, and offer faculty development to ensure that all understand the educational objective and the means chosen to achieve it. Finally, there is a need to assist students as they chart progress towards becoming a professional. |
| **21** | A Schematic Representation of the Professional Identity Formation and Socialization of Medical Students and Residents: A Guide for Medical Educators | Cruess et al | 2015 | Commentary | NIL | Recent calls to focus on identity formation in medicine propose that educators establish as a goal of medical education the support and guidance of students and residents  as they develop their professional identity. Those entering medical school arrive with  a personal identity formed since birth. As they proceed through the educational continuum, they successively develop the identity of a medical student, a resident, and a physician. | NIL | NIL | Drawing on the identity formation and socialization literature, as well as experience gained in teaching professionalism, the authors developed schematic representations of these processes. They adapted them  to the medical context to guide educators as they initiate educational interventions, which aim to explicitly support professional identity formation and the ultimate goal of medical education—to ensure that medical students and residents come to “think, act, and feel like a physician |
| **22** | Medicine as a Community of Practice: Implications for Medical Education | Cruess et al | 2018 | Commentary | NIL | The presence of a variety of independent learning theories makes it difficult for medical educators to construct a comprehensive theoretical framework for medical education, resulting in numerous and often unrelated curricular, instructional, and assessment practices. Linked with an understanding of identity formation, the concept of communities of practice could provide such a framework, emphasizing the social nature of learning. Individuals wish to join the community, moving from legitimate peripheral to full participation, acquiring the identity of community members and accepting the community's norms. | NIL | NIL | Communities of practice can guide the development of interventions to make medical education more effective and can help both learners and educators better cope with medical education’s complexity. An initial step is to acknowledge the potential of communities of practice as the foundational theory. Educational initiatives that could result from this approach include adding communities of practice to the cognitive base; actively engaging students in joining the community; creating a welcoming community; expanding the emphasis on explicitly addressing role modeling, mentoring, experiential learning, and reflection; providing faculty development to support the program; and recognizing the necessity to chart progress toward membership in the community. |
| **23** | Clinical Teachers’ Perceptions of Their Role in Professional Identity Formation | Sternszus et al | 2020 | Qualitative Interviews | 16 | A fundamental goal of medical education is supporting learners in forming a professional identity. While it is known that learners perceive clinical teachers to be critically important in this process, the latter’s perspective is unknown. This study sought to understand how clinical teachers perceive their influence on the professional identity formation of learners. | In 2017, a research assistant conducted 16 semistructured interviews of clinical teachers from 8 specialties at McGill University. The research assistant audiorecorded and subsequently transcribed interviews for analysis. Following principles of qualitative description, the research team developed a coding scheme using both inductive codes (from the words of the participants) and deductive codes (based on the literature and the theory of communities of practice). Through a cross-case analysis, the team then identified salient themes. | Participants struggled to describe their influence on learners’ professional identity without first being prompted to focus on their own identity and its formation. Once prompted, clinical teachers reported viewing their personal and professional identities as integrated and believed that caring for patients was integral to forming their professional identity. They identified explicit role modeling, engaging in difficult conversations, and providing graded autonomy as ways in which they could influence the identity development of learners. However, they had difficulty discerning the magnitude of their influence. | This study was the first to explore professional identity formation from the perspective of clinical teachers. The 2010 Carnegie Foundation report called for an increased focus on professional identity formation. Giving clinical teachers the space and guidance to reflect on this process, helping them make the implicit explicit, and supporting them in using their own experiences as learners to inform their teaching appear to be critical steps in achieving this goal. |
| **24** | Being, becoming, and belonging: reconceptualizing professional identity formation in medicine | Sternszus et al | 2024 | Commentary | NIL | Over the last decade, there has been a drive to emphasize professional identity formation in medical education. This shift has had important and positive implications for the education of physicians. However, the increasing recognition of longstanding structural inequalities within society and the profession has highlighted how conceptualizations of professional identity formation have also had unintended harmful consequences. These include experiences of identity threat and exclusion, and the promotion of norms and values that over- emphasize the preferences of culturally dominant groups. | NIL | NIL | In this paper, the authors put forth a reconceptualization of the process of professional identity formation in medicine through the elaboration of 3 schematic representations. Evolutions in the understandings of professional identity formation, as described in this paper, include re-defining socialization as an active process involving critical engagement with professional norms, emphasizing the role of agency, and recognizing the importance of belonging or exclusion on one’s sense of professional self. The authors have framed their analysis as an evidence- informed educational guide with the aim of supporting the development of identities which embrace diverse ways of being, becoming, and belonging within the profession, while simultaneously upholding the standards required for the profession to meet its obligations to patients and society. |
| **25** | Transformational learning and professional identity formation in postgraduate competency-based medical education | Chow et al | 2024 | Qualitative Interviews | 15 | Residency programmes are in transition to a framework for competency-based medical education (CBME). The intersection of CBME with transformational learning (TL) experiences and professional identity formation (PIF) — particularly within senior learners in transitional states — is unknown but important to understand in order to develop and implement strategies to support trainees' professional development. | Through inductive qualitative methods, we conducted semi-structured interviews (n = 22) of current trainees and recent graduates from adult cardiology residency training programmes within Canada to explore the impact of TL experi- ences on residents' professional growth and identity formation. Interviews were analysed using thematic analysis informed by TL theory. | CBME did not appear to influence trainees' experiences of disorienting dilemmas and TL. Important clinical encounters and interpersonal relationships — in particular, those between mentor and mentee — shaped trainees' professional devel- opment as cardiologists (‘enabling factors’ for TL and PIF). ‘Imposter phenomenon’ was widely prevalent in our sample study population even among graduates who had already completed their training. Requisite elements for transformation (disorienting dilemmas, critical reflection, discourse and action) also contributed to PIF. | TL experiences influenced PIF in senior learners but infrequently intersected with CBME; these experiences were more commonly prompted by disorienting dilemmas relating to clinical outcomes or interpersonal interactions independent of CBME-specific architecture. |
| **26** | A systematic scoping review of mentoring support on professional identity formation | Krishna et al | 2024 | Systematic Review | NIL | Mentoring’s success in nurturing professional identity formation (PIF) has been attributed to its ability to build personalised and enduring mentoring relationships. However, beyond functioning as communities of prac‐ tice (CoPs) supporting socialisation processes, how mentoring integrates programme values and instils a shared iden‐ tity amongst mentees remains unclear. The need for personalised guidance and timely attention to a mentee’s unique needs in evolving mentoring relationships point to the critical role of support mechanisms (‘mentoring support’). We conducted a systematic scoping review (SSR) studying “What is known about mentoring support’s role in nurturing PIF?”. | Adopting PRISMA‐ScR guidelines, this SSR was guided by the Systematic Evidence‐Based Approach (SEBA). Independent searches were carried out on publications featured between 1st January 2000 and 30th June 2023 in PubMed, Embase, ERIC and Scopus databases. The Split Approach saw concurrent, independent thematic and content analyses of the included articles. The Jigsaw Perspective combined complementary themes and catego‐ ries, creating broader themes/categories. The subsequent Funnelling Process formed key domains that platformed the synthesis of the discussion. | Two thousand three hundred forty‐one abstracts were reviewed, 323 full‐text articles were appraised  and 151 articles were included and analysed. The key domains identified were (1) definitions and roles; (2) personalisa‐ tion; (3) shepherding; and (4) PIF. | he success of mentoring in PIF lies in its ability to blend role modelling, supervision, mentoring, coaching and teaching, with self‐care, guided reflection, apprenticeship and assessment to meet the individual needs of the mentee and their changing circumstances. Blending the contents of the mentoring umbrella empha‐ sises the critical role of the mentor and host organisation in supporting mentor training, communications, support |
| **27** | Role modelling in professional identity formation: a systematic scoping review | Koh et al | 2023 | Systematic Review | NIL | Role modelling's pivotal part in the nurturing of a physician's professional identity remains poorly understood. To overcome these gaps, this review posits that as part of the mentoring spectrum, role modelling should be considered in tandem with mentoring, supervision, coaching, tutoring and advising. This provides a clinically relevant notion of role modelling whilst its effects upon a physician's thinking, practice and conduct may be visualised using the Ring Theory of Personhood (RToP). | A Systematic Evidence Based Approach guided systematic scoping review was conducted on articles published between 1 January 2000 to 31 December 2021 in the PubMed, Scopus, Cochrane, and ERIC databases. This review focused on the experiences of medical students and physicians in training (learners) given their similar exposure to training environments and practices. | 12,201 articles were identified, 271 articles were evaluated, and 145 articles were included. Concurrent independent thematic and content analysis revealed five domains: existing theories, definitions, indications, characteristics, and the impact of role modelling upon the four rings of the RToP. This highlights dissonance between the introduced and regnant beliefs and spotlights the influence of the learner's narratives, cognitive base, clinical insight, contextual considerations and belief system on their ability to detect, address and adapt to role modelling experiences. | Role modelling's ability to introduce and integrate beliefs, values and principles into a physician's belief system underscores its effects upon professional identity formation. Yet, these effects depend on contextual, structural, cultural and organisational influences as well as tutor and learner characteristics and the nature of their learner-tutor relationship. The RToP allows appreciation of these variations on the efficacy of role modelling and may help direct personalised and longitudinal support for learners. |
